# Sonic Hedgehog Dependent Phosphorylation by CK1α and GRK2 Is Required for Ciliary Accumulation and Activation of Smoothened

**DOI:** 10.1371/journal.pbio.1001083

**Published:** 2011-06-14

**Authors:** Yongbin Chen, Noriaki Sasai, Guoqiang Ma, Tao Yue, Jianhang Jia, James Briscoe, Jin Jiang

**Affiliations:** 1 Department of Developmental Biology, University of Texas Southwestern Medical Center at Dallas, Dallas, Texas, United States of America; 2 MRC-National Institute for Medical Research, Mill Hill, London, United Kingdom; 3 Markey Cancer Center and Department of Molecular and Cellular Biochemistry, University of Kentucky, Lexington, Kentucky, United States of America; University of Zurich, Switzerland

## Abstract

Hedgehog (Hh) signaling regulates embryonic development and adult tissue homeostasis through the GPCR-like protein Smoothened (Smo), but how vertebrate Smo is activated remains poorly understood. In *Drosophila*, Hh dependent phosphorylation activates Smo. Whether this is also the case in vertebrates is unclear, owing to the marked sequence divergence between vertebrate and *Drosophila* Smo (dSmo) and the involvement of primary cilia in vertebrate Hh signaling. Here we demonstrate that mammalian Smo (mSmo) is activated through multi-site phosphorylation of its carboxyl-terminal tail by CK1α and GRK2. Phosphorylation of mSmo induces its active conformation and simultaneously promotes its ciliary accumulation. We demonstrate that graded Hh signals induce increasing levels of mSmo phosphorylation that fine-tune its ciliary localization, conformation, and activity. We show that mSmo phosphorylation is induced by its agonists and oncogenic mutations but is blocked by its antagonist cyclopamine, and efficient mSmo phosphorylation depends on the kinesin-II ciliary motor. Furthermore, we provide evidence that Hh signaling recruits CK1α to initiate mSmo phosphorylation, and phosphorylation further increases the binding of CK1α and GRK2 to mSmo, forming a positive feedback loop that amplifies and/or sustains mSmo phosphorylation. Hence, despite divergence in their primary sequences and their subcellular trafficking, mSmo and dSmo employ analogous mechanisms for their activation.

## Author Summary

Hedgehog (Hh) signaling governs embryonic development and adult homeostasis in species ranging from *Drosophila* to human, and its malfunction has been implicated in a wide range of human disorders. Hh signal is received by the twelve-transmembrane receptor Patched and transmitted intracellularly by the seven-transmembrane protein Smoothened (Smo). How vertebrate Smo is activated in order to transmit the Hh signal remains poorly understood. Here we investigate the molecular mechanism of mammalian Smo (mSmo) activation and find it is similar to that described for *Drosophila* Smo despite the marked sequence divergence between them. We show that mSmo is activated via phosphorylation at multiple sites by the serine/threonine kinases CK1α and GRK2. We provide evidence that Sonic hedgehog (Shh; the best studied of the three mammalian pathway ligands) can regulate the accessibility of mSmo to these kinases and that phosphorylation promotes the ciliary accumulation of this transmembrane protein in its active conformation. Moreover, increasing concentrations of Shh induce a progressive increase in mSmo phosphorylation that fine-tunes mSmo activity. Thus, our results provide novel insights into the biochemical mechanism of vertebrate Hh signal transduction and reveal a conserved mode of Smo activation.

## Introduction

The Hh family of secreted proteins plays pivotal roles during embryonic development and adult tissue homeostasis [1]–[3]. Aberrant Hh signaling contributes to numerous human disorders including congenital diseases and cancers [4],[5]. In a number of developmental contexts, Hh functions as a morphogen that specifies distinct cell fates in a concentration-dependent manner [1],[2]. For example, in vertebrate neural tube patterning, Shh secreted by the notochord and floor pate forms a ventral to dorsal concentration gradient that specifies distinct pools of neural progenitor cells [6].

Hh exerts its biological function through a signaling cascade that ultimately controls a balance between activator and repressor forms of the Gli family of transcription factors [2]. In the absence of Hh, Gli2 and Gli3 are processed into truncated repressor forms (Gli^R^). Hh signaling blocks Gli processing and converts full-length Gli2/3 into activator forms (Gli^A^). The reception system for the Hh signal consists of a twelve-transmembrane protein Patched (Ptc) as the Hh receptor and a seven-transmembrane protein Smoothened (Smo) as the obligatory Hh signal transducer [2],[3]. Ptc inhibits Smo substoichiometrically through a poorly defined mechanism in the absence of Hh [7]. Binding of Hh to Ptc and the Ihog/Cdo family of proteins alleviates Ptc inhibition of Smo [8]–[14], leading to Smo activation and signal transduction. How Smo is activated and how it transduces the Hh signal to regulate Gli^R^ and Gli^A^ are still poorly understood.

In mammals, Hh signaling depends on the primary cilium, a microtubule-based membrane protrusion found in almost all mammalian cells [15]. Key components in the Hh pathway are found in cilia and exhibit dynamic patterns depending on the Hh signaling state. For example, in the absence of Hh, Ptc localizes to cilia and prevents Smo from accumulating in the cilia; binding of Hh to Ptc triggers reciprocal trafficking of Ptc and Smo, with Ptc moving out of and Smo accumulating in the cilia [16],[17]. Ciliary accumulation of Smo correlates but is not sufficient for Hh pathway activation [16]–[19]. Additional mechanisms, including conformational change in Smo, are likely to be responsible for Smo activation [20]–[22]. Indeed, fluorescence resonance energy transfer (FRET) analysis indicates that both *Drosophila* and mammalian Smo proteins exist as constitutive dimers/oligomers, but in the absence of Hh, Smo C-tails adopt a closed conformation that prevents their association. Hh induces a conformational switch in Smo, leading to dimerization/oligomerization of the C-tails [22]. The mechanisms underlying mammalian Smo ciliary accumulation, conformational change, and activation are largely unknown.

In *Drosophila*, Hh and Ptc reciprocally control Smo cell surface accumulation and conformation through regulating Smo phosphorylation [22]–[25]. In response to Hh, Smo is phosphorylated by protein kinase A (PKA) and casein kinase 1 (CK1) at multiple sites in its C-tail, and these phosphorylation events activate Smo by promoting its cell surface accumulation and active conformation [22],[25]–[27]. However, vertebrate Smo C-tails diverge significantly from that of *Drosophila* Smo and do not contain the PKA/CK1 phosphorylation clusters found in *Drosophila* Smo C-tail [22]. In addition, a systematic mutagenesis study did not reveal any Ser/Thr residues as essential for mammalian Smo activation [28]. These and other observations led to a proposal that mammalian Smo and *Drosophila* Smo are regulated by fundamentally distinct mechanisms [28],[29].

Several studies suggested that G protein coupled receptor kinase 2 (GRK2) positively regulates Shh signaling [30]–[32]. Metabolic labeling experiments revealed that GRK2 is required for the basal phosphorylation of an exogenously expressed Smo [30]. However, it is not clear whether GRK2 directly phosphorylates Smo and how GRK2 activates Shh signaling. In addition, direct evidence for Hh-induced mammalian Smo phosphorylation is lacking. A recent kinome siRNA screen identified CK1α as a positive regulator for Shh signaling, but its mechanism of action remains unknown [33].

In this study, we investigate the activation mechanism of mammalian Smo (henceforth referred to simply as Smo). We demonstrate that Smo is activated via multiple phosphorylation events mediated by CK1α and GRK2 that induce its ciliary accumulation and active conformation. We provide evidence that graded Shh signals induce increasing levels of Smo phosphorylation that fine-tune Smo ciliary localization, conformation, and activity. In addition, we provide evidence that Shh promotes Smo phosphorylation by regulating the accessibility of Smo to its kinases.

## Results

### CK1α and GRK2 Promote Smo Phosphorylation and Conformational Switch

**Figure 1:**
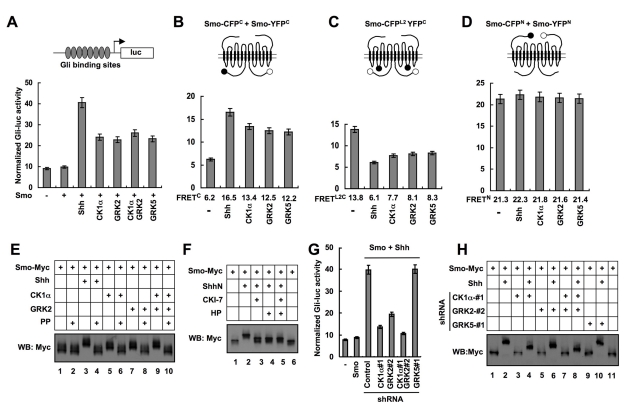
CK1α and GRK2 regulate Smo phosphorylation and conformation. (A) *Gli-luciferase* assay in NIH 3T3 cells transfected with Smo and kinase expressing constructs and treated with or without Shh-conditioned medium. (B–D) FRET analysis in NIH 3T3 cells transfected with Smo-CFP^C^/SmoYFP^C^ (B), SmoCFP^L2^YFP^C^ (C), or SmoCFP^N^/SmoYFP^N^ (D) and treated with or without Shh-conditioned medium or cotransfected with indicated kinase expressing constructs. Filled and open circles in the cartoons denote CFP and YFP, respectively. (E–F) Cell extracts were prepared from NIH 3T3 cells transfected by indicated constructs and treated with or without indicated reagents, separated on SDS-PAGE gel containing 25 mM Phos tag-conjugated acrylamide, followed by immunoblotting with an anti-Myc antibody. The inhibitors used are: CK1 inhibitor, CKI-7 (10 µM); GRK inhibitor Heparin (HP, 1 µM). PP indicates λ-phosphatase treatment. (G) *Gli-luc* assay in NIH 3T3 cells stably expressing shRNA targeting CK1α, GRK2, or GRK5 and transfected with or without Smo and treated with or without Shh-conditioned medium. (H) Cell extracts were prepared from NIH 3T3 cells stably expressing shRNA targeting CK1α, GRK2, or GRK5 and transfected with Smo-Myc and treated with or without Shh-conditioned medium, separated on the Phos tag-conjugated SDS-PAGE gel and immunoblotted with an anti-Myc antibody.

A previous study revealed that CK1α siRNA blocked Shh pathway activation in C3H10T1/2 cells [33]. To determine how CK1α positively regulates Shh signaling, we tested whether CK1α activates Smo. Coexpression of CK1α with Smo in NIH3T3 cells activated a *Gli-luciferase* (*Gli-luc*) reporter gene, although the fold of activation was less dramatic compared with Shh stimulation (Figure 1A). In line with a previous finding [31], coexpression of GRK2 with Smo also activated *Gli-luc* in NIH3T3 cells (Figure 1A). Coexpression of CK1α and GRK2 with Smo had a slightly stronger effect on *Gli-luc* expression than overexpression of each kinase alone (Figure 1A). Overexpression of another GRK family member (GRK5) with Smo activated the *Gli-luc* reporter gene similarly to GRK2 (Figure 1A), indicating that overexpressed GRK5 and GRK2 have a similar activity in the Shh pathway.

We also examined the effect of CK1α/GRK2 overexpression on *Gli-luc* expression in the absence of exogenously expressed Smo. Consistent with previous findings [31],[33], overexpression of CK1α, GRK2, or both only slightly increased the expression of *Gli-luc* reporter gene (Figure S1G). Thus, CK1α/GRK2 overexpression synergized with Smo overexpression to drive Shh pathway activation.

Our previous FRET analysis indicated that Shh induces a conformational change in Smo from a closed to an open conformation [22]. In the closed conformation, Smo exists as a dimer/oligomer through an N-terminal interaction(s), which results in high basal FRET between CFP and YFP fused to the N-termini of two Smo molecules (FRET^N^); however, Smo C-tail folds back and is in close proximity to the intracellular loops, resulting in high intramolecular FRET between CFP inserted in the second intracellular loop (L2) and YFP fused to the C-terminus (FRET^L2C^) and low intermolecular FRET between CFP and YFP fused to the C-termini of two Smo molecules (FRET^C^) (Figure 1B–D) [22]. Shh induced a marked decrease in FRET^L2C^ and a concomitant increase in FRET^C^ without affecting FRET^N^ (Figure 1B–D) [22], suggesting that Smo C-tails move away from the intracellular loops and form dimers/oligomers. To determine whether CK1α and GRK regulate Smo conformation, we carried out FRET analysis using the Smo biosensors indicated in Figure 1B–D. We found that overexpression of CK1α, GRK2, or GRK5 resulted in a significant increase in FRET^C^ (Figure 1B) and a marked decrease in FRET^L2C^ (Figure 1C). In contrast, overexpression of these kinases did not cause a significant change in FRET^N^ (Figure 1D). These results suggest that excessive CK1α and GRK2/5 kinase activities induce a conformational change in Smo similar to that induced by Shh stimulation.

Having established that CK1α and GRK2 act upstream of Smo, we then determined whether CK1α and GRK2 could promote Smo phosphorylation using a Phos-tag gel that specifically retards phosphorylated proteins [34]. We found that coexpression of CK1α, GRK2, or both with a Myc-tagged Smo (Smo-Myc) resulted in a clear mobility shift of Smo-Myc on Phos-tag PAGE that was abolished by phosphatase treatment (Figure 1E, lanes 5–10), suggesting that CK1α and GRK2 can promote Smo phosphorylation.

### CK1α and GRK2 Are Required for Shh-Induced Smo Phosphorylation

We next determined whether Shh normally induces Smo phosphorylation and whether it does so through CK1α and GRK2. Treating Smo-Myc transfected cells with a Shh-conditioned medium but not a control medium induced a marked mobility shift of Smo-Myc that was abolished by phosphatase treatment (Figure 1E, lanes 3–4). Importantly, Shh-induced Smo-Myc mobility shift was greatly reduced by treating cells with a CK1 inhibitor CKI-7 [35] and/or a GRK inhibitor heparin [36] (Figure 1F), suggesting that Shh induces Smo phosphorylation through CK1 and GRK kinase activities.

To establish that CK1α and GRK2 are required for Shh-induced Smo phosphorylation, we generated cell lines stably expressing shRNA targeting CK1α, GRK2, or GRK5. Two independent shRNA constructs that effectively and selectively knocked down the targeted kinase were employed in our assay (Figure S1A–B). In line with previous findings [31]–[33], CK1α or GRK2 shRNA inhibited Shh pathway activity in the *Gli-luc* reporter assay (Figure 1G, Figure S1C–D, H). In contrast, GRK5 shRNA did not alter Shh-induced *Gli-luc* expression (Figure 1G, Figure S1E). Importantly, CK1α and/or GRK2 shRNA but not GRK5 shRNA reduced Shh-induced mobility shift of Smo-Myc (Figure 1H, Figure S1F), suggesting that CK1α and GRK2 are required for Shh-induced Smo phosphorylation. We note that Shh-induced Smo mobility shift was not completely abolished by silencing CK1α and GRK2, likely due to an incomplete elimination of these kinase activities by the RNAi approach (Figure S1B). However, it is also possible that the residual Smo-Myc phosphorylation in the presence of CK1α and GRK2 shRNA could be due to the involvement of another kinase(s).

### CK1 and GRK Phosphorylate Smo C-tail at Multiple Sites

**Figure 2:**
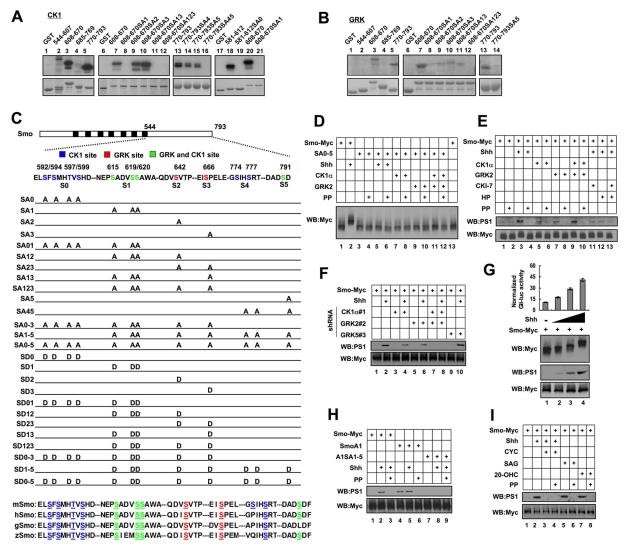
CK1 and GRK phosphorylate multiple sites in Smo C-tail. (A–B) In vitro kinase assay using a recombinant CK1δ or GRK5 and purified GST-fusion proteins carrying indicated Smo fragments. (C) A schematic drawing of a full-length Smo with the sequences of the six CK1/GRK phosphorylation sites shown underneath (S0–S5) and color coded: blue for CK1 specific sites; red for GRK specific sites; green for sites phosphorylated by both CK1 and GRK. Smo variants with the indicated substitutions are listed. Sequence alignment shows that the CK1/GRK phosphorylation sites are highly conserved among Mouse (m), Human (h), Chick (g), and Zebrafish (z) Smo proteins. (D) Cell extracts were prepared from NIH 3T3 cells transfected with Smo-Myc or SmoSA0–5-Myc and with or without cotransfection of the indicated kinase expressing constructs and treated with or without Shh-conditioned medium. The extracts were separated on Phos tag-conjugated SDS-PGAE gel and immunoblotted with an anti-Myc antibody. PP indicates λ-phosphatase treatment. (E) Cell extracts were prepared from NIH 3T3 cells transfected with Smo-Myc and with or without indicated kinase expressing constructs, followed by treating with or without Shh-conditioned medium or indicated kinase inhibitors. The extracts were subjected to SDS-PAGE, followed by immunoblotting with the PS1 antibody. The membrane was stripped and probed with Myc antibody for Smo-Myc. (F) Cell extracts prepared from shRNA lines targeting CK1α, GRK2, or GRK5 transfected by Smo-Myc and treated with or without Shh-conditioned medium were analyzed as in (E). (G) NIH 3T3 cells were transiently transfected with Smo-Myc and treated without or with increasing levels of ShhN peptides (1 nM, 2 nM, 5 nM). *8XGliBS-luc* activities were normalized by control *pRL-TK*. Cell extracts were separated on Phos tag-conjugated (top panel) or regular SDS-PAGE gel (bottom two panels), followed by immunoblotting with Myc or PS1 antibody. (H–I) Cell extracts from NIH 3T3 cells transfected with the indicated constructs and treated with or without Shh-conditioned medium, CYC (cyclopamine; 10 µM), SAG (200 nM), or 20-OHC (20α-hydroxycholesterol; 10 µM) were separated on SDS-PAGE gel and probed with PS1 antibodies. The membranes were stripped and probed with Myc antibody to monitor Smo-Myc levels.

To determine whether CK1 and GRK directly phosphorylate Smo, we developed an in vitro kinase assay in which purified GST-fusion proteins containing different regions of Smo C-tail were incubated with a recombinant CK1 (CK1δ from New England Biolabs) or GRK (GRK5 from Cell Signaling Technology) in the presence of γ^32^-p-ATP. Two non-overlapping fragments, amino acid (aa) 608–670 and aa 770–793, were phosphorylated by both CK1 and GRK (Figure 2A, lanes 3, 5; Figure 2B, lanes 3, 5), suggesting that they harbor CK1 and GRK sites. GRK family kinases tend to phosphorylate S/T in an acidic environment [37]. aa 608–670 contains three sequences (EPS_615_ADVS_619_S_620_A, QDVS_642_VT, and EIS_666_PELE) and aa 770–793 contains one sequence (DADS_791_DF) that match GRK consensus sites (Figure 2C). Indeed, mutating S_615_, S_619_ and S_620_ (SA1), S_642_ (SA2), or S_666_ (SA3) reduced and their combined mutations (SA123) abolished phosphorylation of aa 608–670 (Figure 2B, lanes 8–12; Figure S2A and S2C, lanes 3–9), whereas mutating S_791_ abolished phosphorylation of aa 770–793 by GRK (Figure 2B, lane 14), suggesting that these Ser residues are GRK sites.

CK1 phosphorylation sites conform to the consensus: D/E/S/T(P)X_1–3_S/T [38]. Site-directed mutagenesis revealed that S_615_, S_619_, and S_620_ mediated CK1 phosphorylation of aa 608–670 (Figure 2A, lanes 8–12; Figure S2A and Figure S2B, lanes 3–9), whereas S_774_, S_777_, and S_791_ mediated CK1 phosphorylation of aa 770–793 (Figure 2A, lanes 14–16). aa 608–670SA1, which has S_615_, S_619_, and S_620_ mutated to Ala but contains intact S_642_ and S_666_, was not phosphorylated by CK1 (Figure 2A, lane 8), suggesting that S_642_ and S_666_ are not CK1 sites. In addition, we found that aa 581–612 was phosphorylated by CK1 but not by GRK (Figure 2A, lane 18; unpublished data). This region contains a sequence matching CK1 consensus sites: ELS_592_FS_594_MHT_597_VS_599_. Indeed, mutating S_592_, S_594_, T_597_, and S_599_ to Ala (aa 581–612SA0) abolished CK1 phosphorylation of aa 581–612 (Figure 2A, lane 19).

For simplicity, we referred to S_592_, S_594_, T_597_, and S_599_ collectively as S0; S_615_, S_619_, and S_620_ as S1; S_642_ as S2; S_666_ as S3; S_774_ and S_777_ as S4; and S_791_ as S5 (Figure 2C). Thus, S1 and S5 are phosphorylation sites for both CK1 and GRK, whereas S0/S4 and S2/S3 are selectively phosphorylated by CK1 and GRK, respectively. Sequence alignment indicates that these phosphorylation sites are conserved among vertebrate Smo proteins (Figure 2C).

To determine if the CK1/GRK sites identified in vitro mediate Shh-induced Smo phosphorylation in vivo, we mutated S0–S5 to Ala in Smo-Myc (SA0–5, Figure 2C). We found that the SA0–5 mutation abolished Shh, CK1α, or GRK2-induced Smo mobility shift (Figure 2D, lanes 5–12). Furthermore, CK1α and GRK2 neither activated SA0–5 nor induced its conformational change (Figure S2D–E).

To further characterize Smo phosphorylation in vivo, we attempted to generate phospho-specific antibodies against phosphorylated CK1/GRK sites and succeeded in obtaining an antibody (PS1) that specifically recognizes phosphorylated S1 (pS_615_, pS_619_, and pS_620_, Figure S2F). To monitor phosphorylation at S1, NIH3T3 cells were transfected with Smo-Myc and stimulated with or without Shh-conditioned medium. In the absence of Shh, Smo-Myc exhibited a weak PS1 signal likely due to basal phosphorylation (Figure 2E, lane 1). Shh induced a clear increase in the intensity of the PS1 signal (Figure 2E, lane 3). Coexpression of CK1α, GRK2, or both also increased the PS1 signal (Figure 2E, lanes 5, 7, and 9). On the other hand, Shh-stimulated S1 phosphorylation was diminished by treating cells with the CK1 and/or GRK2 kinase inhibitors (Figure 2E, lanes 11–13). Furthermore, CK1α or GRK2 shRNA reduced whereas combined CK1α/GRK2 shRNA nearly abolished S1 phosphorylation (Figure 2F, lanes 4, 6, and 8; Figure S2G). In contrast, GRK5 shRNA did not affect S1 phosphorylation (Figure 2F, lane 10; Figure S2G), consistent with its lack of effect on Shh pathway activity. These results demonstrate that Shh induces S1 phosphorylation by CK1α and GRK2.

### Regulation of Smo Phosphorylation by Graded Shh Signals, Oncogenic Mutations, and Small Molecules

Hh signaling strength depends on the level of Hh ligand [2]. To determine if the level of Shh pathway activity correlates with the level of Smo phosphorylation, NIH3T3 cells transfected with Smo-Myc were treated with different levels of Shh, followed by mobility shift assay on the phospho-tag gel or western blot with PS1. We found that increasing levels of Shh induced a progressive increase in the degree of Smo-Myc mobility shift (Figure 2G), suggesting that Smo-Myc was phosphorylated at more sites in response to higher levels of Shh. In addition, we found that increasing levels of Shh resulted in a gradual increase in the PS1 signal (Figure 2G), suggesting that the frequency of S1 phosphorylation increases with increasing Shh concentration.

Several oncogenic mutations in human Smo have been identified, including M1 and M2 [39]. The M2 mutation occurs in the seventh transmembrane domain whose murine counterpart is the A1 mutation [20],[39]. Previous studies suggest that SmoA1 exhibits constitutive activity, accumulates at primary cilia, and adopts an open conformation [16],[20],[22]. We found that SmoA1 exhibited slower mobility and elevated PS1 signal intensity regardless of Shh treatment (Figure 2H, lanes 4–5; Figure S2H) and that A1-induced PS1 signal and mobility shift were abolished by the S1–5 mutation (A1SA1–5) (Figure 2H, lanes 7–8; Figure S2H), suggesting that the oncogenic mutation mimics Shh stimulation to induce Smo phosphorylation at CK1/GRK sites. We also observed that M1 increased Smo phosphorylation (see below).

Previous studies demonstrated that small molecules including SAG and 20α-hydroxycholesterol (20-OHC) promote whereas cyclopamine blocks Smo activation [20],[21],[40],[41]. We found that SAG and 20-OHC induced whereas cyclopamine blocked Smo phosphorylation at CK1/GRK sites (Figure 2I), suggesting that these small molecules regulate Shh signaling at the level of Smo phosphorylation.

### CK1/GRK Phosphorylation Sites Are Essential for Smo Activation

**Figure 3:**
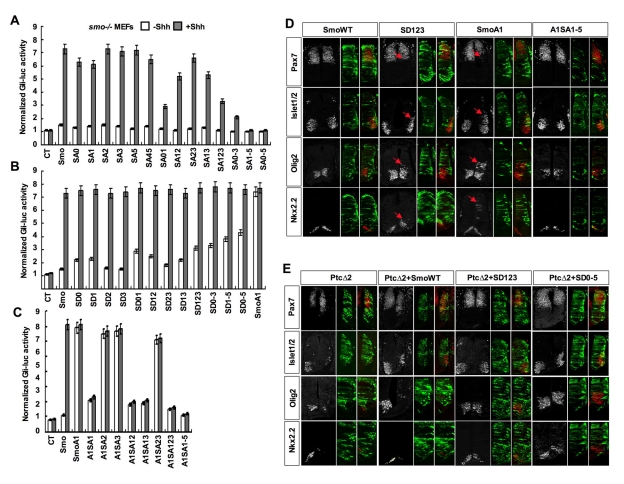
Phosphorylation at multiple CK1 and GRK sites regulates Smo activity both in vitro and in vivo. (A–C) *Gli-luc* activity assays for *smo*−*/*− MEFs transfected with the indicated Smo expressing constructs together with the *8XGliBS-luc* reporter gene and a control *pRL-TK*. The cells were treated with or without Shh-conditioned medium 2 d after transfection. (D–E) HH st11–12 chick neural tubes were electroporated with the indicated expression constructs and assayed by immunohistochemistry 48 h after transfection. (D) The expression of Pax7, Islet1/2, Olig2, and Nkx2.2 in anterior thoracic regions of embryos transfected with wild-type Smo (SmoWT), SmoSD123, SmoA1, or SmoA1SA1–5. Cells expressing the constructs were identified by CFP (green). Arrows indicate the expanded expression of Islet1/2, Olig2, and Nkx2.2 and the reduction of Pax7 expression. SmoA1 exhibited potent constitutive activity, leading to the repression of Pax7 and induction of ectopic Islet1/2, Olig2, and Nkx2.2. Mutating multiple CK1/GRK sites in SmoA1 (A1SA1–5) diminished its constitutive activity. SmoSD123 also exhibited constitutive activity, resulting in a reduction of Pax7 expression and expansion of Islet1/2, Olig2, and Nkx2.2, albeit less dramatic than SmoA1. Note that there was also a subtle expansion of Olig2 in some embryos transfected with SmoWT. (E) Embryos co-transfected at a ratio of 2∶1 with Ptc1^Δloop2^ (PtcΔ2) and either Smo-WT, SmoSD0–5, or SmoA1 and assayed for Pax7, Islet1/2, Olig2, and Nkx2.2 expression. Ptc1^Δloop2^ inhibits Shh signaling resulting in the repression of Islet1/2, Olig2, and Nkx2.2, and a ventral expansion of Pax7. SmoSD123 and SmoSD0–5 but not SmoWT overcame the dominant inhibitory effect of Ptc1^Δloop2^.

To determine the functional significance of Smo phosphorylation, CK1/GRK sites were mutated to Ala individually or in different combinations (referred to as SA mutation; Figure 2C), and the effect of SA mutations on Smo activity was determined by the *Gli-luc* reporter assay in *smo*−/− MEFs. Mutating S0 (SA0) or S1 (SA1) slightly reduced whereas their combined mutations (S01) markedly inhibited Shh-induced Smo activity (Figure 3A). While mutating S2 to S5 either individually (SA2, SA3, SA5) or in combinations (SA23, SA45) had little if any effect on Smo activation (Figure 3A), combined mutations of these sites with S1/S0 (SA12, SA13, SA123, SA0–3, SA1–5, SA0–5) resulted in a progressive decrease in Shh-induced Smo activity (Figure 3A). Finally, simultaneously mutating all CK1/GRK sites (SA0–5) completely abolished Shh-induced Smo activation. These results suggest: 1) phosphorylation at CK1/GRK sites is essential for Smo activation; 2) S0 and S1 are the major sites while S2 to S5 may play a fine-tuning role; and 3) the level of Smo activity correlates with its level of phosphorylation.

To determine whether phosphorylation renders constitutive Smo activity, we converted individual or different combinations of CK1/GRK sites to Asp (referred to as SD mutations) to mimic different levels of phosphorylation (Figure 2C). SD mutations of individual sites (SD0, SD1, SD2, SD3) or several combinations (SD23, SD12, SD13) caused little if any increase in the basal activity of Smo (Figure 3B); however, other combinations (SD01, SD123, SD0–3, SD1–5, SD0–5) resulted in a clear elevation of Smo basal activity and the level of basal activity correlates with the number of altered sites (Figure 3B). Nevertheless, the constitutive activities of SmoSD variants are lower compared with that of SmoA1 (Figure 3B). Furthermore, the SD variants were further stimulated by Shh, whereas SmoA1 was no longer regulated by Shh (Figure 3B). Thus, phosphorylation at CK1/GRK sites increases Smo activity in a dose dependent manner but does not confer full activation.

To determine whether the oncogenic mutation activates Smo through its phosphorylation, we mutated several CK1/GRK sites to Ala in SmoA1. Mutating S2/3 (A1SA2, A1SA3, A1SA23) had little if any effect on SmoA1 activity (Figure 3C). In contrast, S1 mutation (A1SA1) or combined mutations of S1 with other sites (A1SA12, A1SA13, A1SA123, A1SA1–5) greatly reduced or nearly abolished SmoA1 activity (Figure 3C), suggesting that S1 phosphorylation is critical for the oncogenic mutation to activate Smo. Of note, the SA1 mutation had a more profound effect on the activity of SmoA1 than that of wild type Smo in the presence of Shh (compare SmoA1SA1 with SmoSA1+Shh). The reason for this difference is unclear, but it is possible that the oncogenic mutation may not fully mimic Shh stimulation so that SmoA1 relies on S1 phosphorylation for its activation more than wild type Smo.

We also found that mutating SA0–5 abrogated SAG-induced Smo activation, whereas SD0–5 exhibited resistance to cyclopamine inhibition (Figure S2I), suggesting that SAG and cyclopamine regulate Smo activity by influencing its phosphorylation.

### Mutating CK1/GRK Sites Affects Smo Activity In Vivo

We next used chick neural tubes to determine the role of Smo phosphorylation in Shh signaling in living organisms. CFP-tagged constructs expressing wild type (WT) or mutant forms of Smo were electroporated into one side of the neural tube, leaving the other side as an internal control, followed by immunostaining to visualize the expression of various Hh responsive genes. Electroporation of SmoWT or Smo variants that mimic low-level phosphorylation (SD1, SD12) did not significantly alter the expression of the marker genes (Figure 3D and Figure S3A); however, electroporation of Smo variants that mimic high-level phosphorylation (SD123, SD1–5, SD0–5) resulted in a dorsal expansion of several ventral markers, including Nkx2.2, Olig2, Nkx6.1, and Islet1 (Figure 3D and Figure S3D). Furthermore, SD123 and SD0–5 but not SmoWT restored the expression of ventral markers suppressed by a dominant form of Ptc, Ptc1^Δloop2^ (PtcΔ2), as well as prevented the derepression of the dorsal marker Pax7 (Figure 3E) [42]. These results suggest that phosphorylation at CK1/GRK sites increased the basal activity of Smo in the chick neural tubes.

In line with tissue culture experiments, SmoA1 is more potent than SmoSD variants in inducing ectopic expression of ventral marker genes in chick neural tubes (Figure 3D, Figure S3B). Mutating S1 (A1SA1) or combination of S1 with other sites to Ala (A1SA12, A1SA13, A1SA123, A1SA1–5) diminished or completely abolished A1-mediated ectopic activation of ventral markers or suppression of Pax7, whereas mutating S2 and 3 (A1S23) had little if any effect on SmoA1 activity (Figure 3D, Figure S3B), suggesting that phosphorylation at S1 is critical for the oncogenic mutation to activate Smo in the chick neural tube.

### Phosphorylation of Smo Promotes Its Ciliary Accumulation

**Figure 4:**
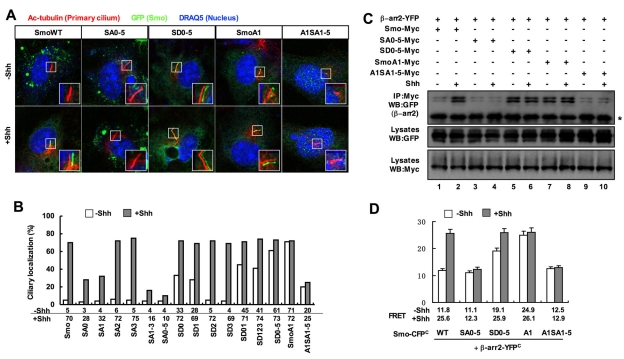
Regulation of Smo ciliary localization by CKI/GRK-mediated phosphorylation. (A–B) Wild-type MEFs infected with retrovirus encoding CFP-tagged wild-type Smo or indicated Smo variants and treated with or without Shh-conditioned medium were immunostained to show the expression of Acetylated (Ac)-tubulin (Red) that labels the primary cilium, GFP (green) that labels the CFP-tagged Smo proteins, and DRAQ5 (blue) that labels the nucleus (A). The insets show enlarged views of the selected regions with shifted overlays. Quantification of ciliary localization of infected Smo variants as indicated by the percentage of GFP+ cilia is shown in (B). Over 100 ciliated cells were counted for each Smo construct. (C) Shh promotes the interaction between Smo with β-arr2. NIH 3T3 cells were transfected with the indicated Myc-tagged Smo variants and YFP tagged β-arr2. Cells lysates were subjected to western blot analysis with Myc and GFP antibodies or immunoprecipitated with the Myc antibody, followed by western blot analysis with the GFP antibody. Asterisk indicates IgG heavy chain. (D) FRET analysis of NIH 3T3 cells transfected with indicated C-terminally CFP-tagged Smo variants and C-terminally YFP-tagged β-arr2 and treated with or without Shh-conditioned medium.

Shh induces ciliary accumulation of Smo that correlates with pathway activation, but the underlying mechanism is poorly understood [16],[17]. We determined whether Shh promotes Smo ciliary localization by inducing its phosphorylation at CK1/GRK sites by examining ciliary localization of CFP-tagged wild type or phosphorylation site mutant forms of Smo in MEF cells treated with or without Shh-conditioned medium. As overexpression by transient transfection caused high basal ciliary localization of Smo, we used retroviral infection to express low levels of exogenous Smo. In these conditions, SmoWT was found in less than 5% of cilia in the absence of Shh but accumulated in ∼70% of cilia in response to Shh treatment (Figure 4A–B). We found that SA mutations inhibited Shh-induced whereas SD mutations promoted basal ciliary accumulation of Smo in a dose-dependent manner (Figure 4A–B). In addition, constitutive ciliary localization of SmoA1 was inhibited by the SA1–5 mutation (A1SA1–5, Figure 4A–B). Thus, phosphorylation at CK1/GRK sites is both necessary and sufficient for the ciliary localization of Smo.

A recent study suggested that β-arrestins mediate Smo ciliary localization by binding to Smo and facilitating its interaction with the kinesin-II motor [43]. We hypothesized that Shh-induced Smo phosphorylation promotes its ciliary localization by recruiting β-arrestins. To test this possibility, we transfected NIH 3T3 cells with a YFP-tagged β-arrestin2 (β-arr2-YFP) together with Myc-tagged wild type or mutant forms of Smo. As shown in Figure 4, both Shh and the A1 mutation increased the amount of β-arr2 coimmunoprecipitated with Smo (Figure 4C, lanes 2, 7). The SA mutations nearly abolished Shh- or A1-stimulated interaction (Figure 4C, lanes 4,10), whereas SD0–5 promoted Smo/β-arr2 interaction (Figure 4C, lane 5).

We also confirmed that phosphorylation regulates Smo/β-arr2 association using FRET assay. We found that Shh and A1 increased the FRET between Smo-CFP and β-arr2-YFP, and this increase was abolished by the SA0–5 mutation (Figure 4D). Conversely, SD0–5 increased the basal FRET between Smo-CFP and β-arr2-YFP. Thus, Shh-induced phosphorylation at CK1/GRK sites increases the association between Smo and β-arr2, which may account for the increased ciliary localization of Smo.

### Phosphorylation Promotes an Open Conformation of Smo

**Figure 5:**
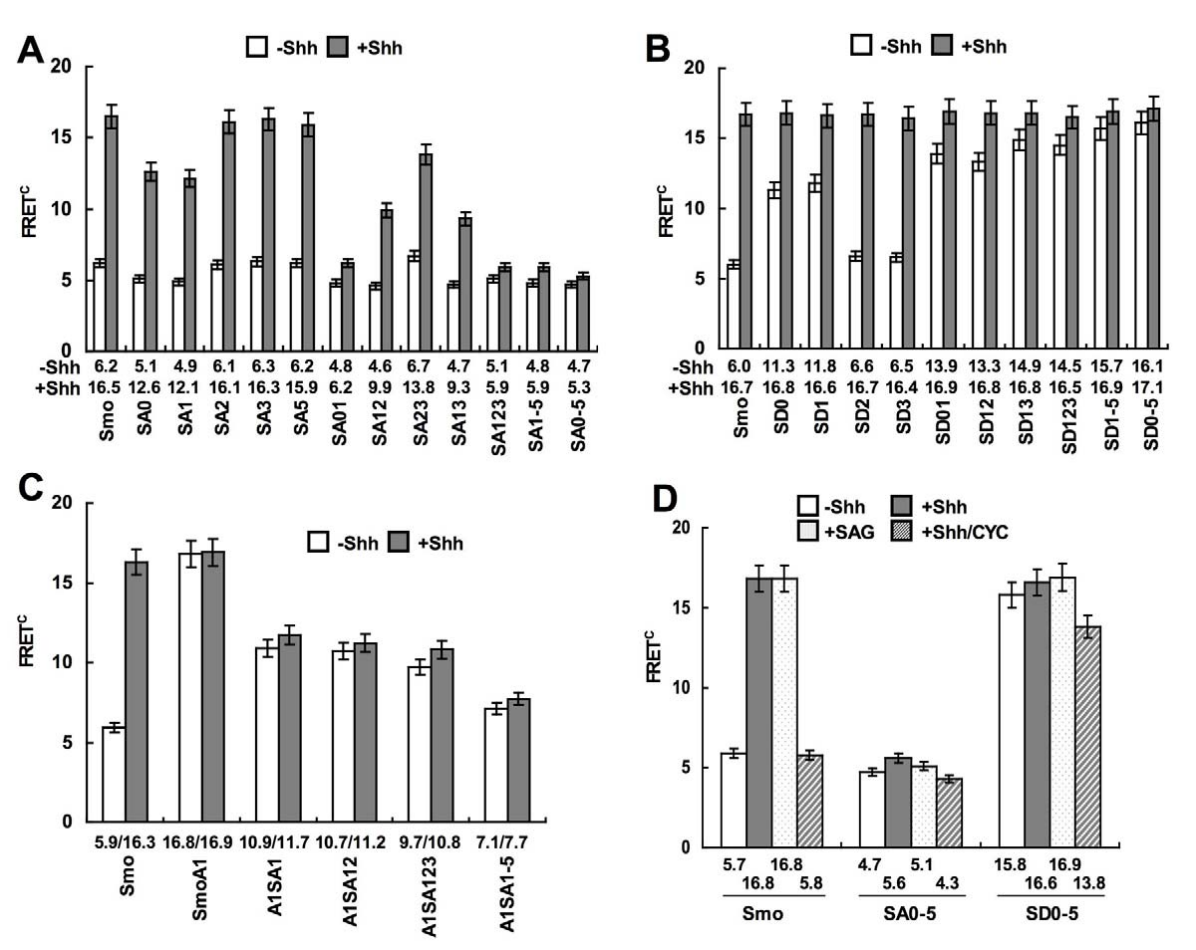
Shh, A1, SAG, and cyclopamine regulate Smo conformation through CK1/GRK-mediated phosphorylation. (A–C) FRET analyses of NIH 3T3 cells transfected with C-terminally CFP/YFP-tagged wild type Smo or Smo variants with the indicated mutations and treated with or without Shh-conditioned medium (mean ± s.d., *n*≥10). (D) FRET analysis in NIH 3T3 cells transfected with Smo-CFP^C^/YFP^C^, SmoSA0–5-CFP^C^/YFP^C^, or SmoSD0–5-CFP^C^/YFP^C^, and treated without or with Shh-conditioned medium, 200 nM SAG, or a combination of Shh-conditioned medium and 10 µM cyclopamine (mean ± s.d., *n*≥10).

To determine whether phosphorylation at CK1/GRK sites regulates Smo conformation, we mutated individual or combination of CK1/GRK sites to either Ala or Asp in C-terminally CFP/YFP-tagged Smo and carried out FRET analysis in NIH3T3 cells. SA0 or SA1 slightly reduced Shh-induced FRET^C^, whereas individual mutations at other sites (SA2, SA3, SA5) had no effect (Figure 5A). S0 and S1 double mutation (S01) or combined mutation of S0/1with other sites (SA12, SA13, SA123, SA1–5, SA0–5) greatly reduced or nearly abolished Shh-induced FRET^C^ (Figure 5A). On the other hand, the SD mutations resulted in a dose-dependent increase in the basal FRET^C^ (Figure 5B). Overall, the effects of SA or SD mutations on Shh-induced FRET^C^ correlated with their effects on Shh-induced Smo activation.

The SA mutations also diminished A1-induced FRET^C^ (Figure 5C). Furthermore, SA0–5 abolished SAG-induced FRET^C^, whereas SD0–5 conferred high basal FRET^C^ even in the presence of cyclopamine (Figure 5D). Thus, phosphorylation at CK1/GRK sites induced by Shh, A1, and SAG causes a conformational switch in Smo C-tail, leading to its dimerization, whereas cyclopamine locks Smo in the closed conformation by blocking its phosphorylation.

### Smo Phosphorylation at the Primary Cilium

**Figure 6:**
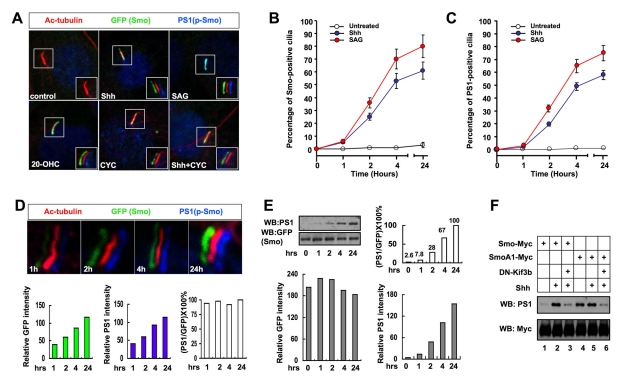
Smo phosphorylation at the primary cilium. (A) NIH 3T3 cells stably expressing Smo-CFP were either untreated (control) or treated with Shh-conditioned medium (Shh), SAG (200 nM), 20-OHC (10 µM), CYC (10 µM), or a combination of Shh-conditioned medium and CYC (10 µM). Cells were immunostained to show the expression of acetylated tubulin (red; primary cilium), GFP (green; Smo), and PS1 (phosphorylated Smo; blue). Images in the insets are shifted overlays of the selected fields. (B–C) The percentage of Smo-CFP (GFP) or phosphorylated Smo (PS1) positive primary cilia at different time after cells were treated with or without Shh or SAG (200 nM). Over 100 ciliated cells were counted for each time point, *n* = 3. (D) NIH 3T3^Smo-CFP^ cells were treated with 200 nM SAG for 1, 2, 4, or 24 h, followed by immunostaining to show the expression of acetylated tubulin (red), GFP (green), and PS1 (blue). Representative images with shifted overlay were shown for each time point. Histograms underneath show the relative intensities of PS1 or GFP fluorescence signals and their ratios (PS1/CFP) at each time point. The ratios are normalized to that of 24 h time point, which is set at 100%. (E) NIH 3T3^Smo-CFP^ cells were treated with Shh-conditioned medium for 0, 1, 2, 4, or 24 h. Cell extracts at each time point were separated on SDS-PAGE and probed with the indicated antibodies. Histograms show the relative intensities of PS1 and GFP bands quantified by the ImageJ software and their ratio (PS1/CFP) normalized to that at 24 h. (F) Cell extracts prepared from NIH 3T3 cells transfected with the indicated constructs and treated with or without Shh-conditioned medium were subjected to western blot analysis with the indicated antibodies.

To examine the spatial and temporal regulation of Smo phosphorylation, we carried out immunohistochemistry experiments using the PS1 antibody that recognizes phosphorylated S1. Because PS1 failed to detect endogenous Smo, we generated NIH3T3 cells stably expressing low levels of Smo-CFP (NIH3T3^Smo-CFP^). NIH3T3^Smo-CFP^ did not exhibit significant basal ciliary localization of Smo-CFP or ciliary PS1 signal but accumulated both signals in the cilia upon stimulation with Shh, SAG, or 20-OHC (Figure 6A, Figure S4A). Cyclopamine induced ciliary accumulation of Smo-CFP but not PS1 (Figure 6A, Figure S4A). Furthermore, cyclopamine blocked Shh or 20-OHC but not SAG-induced ciliary PS1 signals (Figure 6A; Figure S4A). Thus, Shh, SAG, and 20-OHC induced ciliary accumulation of phosphorylated Smo, whereas cyclopamine trapped unphosphorylated Smo in the cilia. The difference in the sensitivity of SAG and 20-OHC to cyclopamine could be due to different mechanisms of action employed by these small molecules to regulate Smo.

To examine the dynamics of Smo phosphorylation, we treated NIH3T3^SmoCFP^ cells with Shh-conditioned medium or SAG for different periods of time (1, 2, 4, and 24 h). In line with a previous report [17], both Shh and SAG induced a rapid ciliary accumulation of Smo-CFP, and the percentage of Smo-CFP positive cilia as well as the mean intensity of SmoCFP signal increased over time (Figure 6B–D). Importantly, we observed a similar kinetics for PS1 accumulation in the primary cilia (Figure 6B–D). Furthermore, the ratio of PS1 versus Smo-CFP signal intensity in primary cilia remained relatively constant over time.

We also monitored Smo phosphorylation in whole cells by western blot using the PS1 and GFP antibodies. We found that the ratio of PS1 versus Smo-CFP signal intensity was lower at early time points and gradually increased over time (Figure 6E). Thus, Smo phosphorylation exhibited faster kinetics in primary cilia than in whole cells, implying that Smo could be preferentially phosphorylated near or in the primary cilia in response to Shh or SAG, leading to its rapid accumulation in the cilia.

### Efficient Smo Phosphorylation Depends on the Kinesin-II Ciliary Motor

To investigate whether primary cilia regulate Smo phosphorylation, we disrupted the cilia using a dominant negative form of Kif3b (DN-Kif3b), a subunit of the kinesin-II motor required for cilia formation [44]. We found that DN-Kif3b diminished but did not completely block Shh-induced PS1 signal associated with either Smo-Myc or SmoA1-Myc (Figure 6F, lanes 3, 6), suggesting that efficient phosphorylation at S1 depends on the kinesin-II ciliary motor.

We also analyzed whether the primary cilium is required for Shh-induced Smo conformational change by measuring FRET^C^ in the wild type or *Kif3a*−/− MEFs transfected with wild type or mutant forms of Smo-CFP^C^/YFP^C^. We found that Shh or A1-induced FRET^C^ was dramatically reduced in *Kif3a*−*/*− MEFs compared with WT MEFs (Figure S4B). In contrast, SmoSD0–5-CFP^C^/YFP^C^ exhibited high FRET^C^ in both WT and *Kif3a*−*/*− MEFs (Figure S4B). Thus, in the absence of primary cilia, Shh and A1 failed to induce the active Smo conformation because of compromised Smo phosphorylation, but an open conformation can be restored by phospho-mimetic mutations.

Although SmoSD0–5 adopts an open conformation in *Kif3a*−*/*− MEFs, it failed to induce any *Gli-luc* expression in the absence of primary cilia (Figure S4C). In contrast, overexpression of Gli1 in *Kif3a*−*/*− MEFs activated the *Gli-luc* reporter. These observations suggest that the primary cilium is not only required for Smo activation but is also essential for signal transduction downstream of activated Smo.

### Shh Promotes Binding of CK1α and GRK2 to Smo

**Figure 7:**
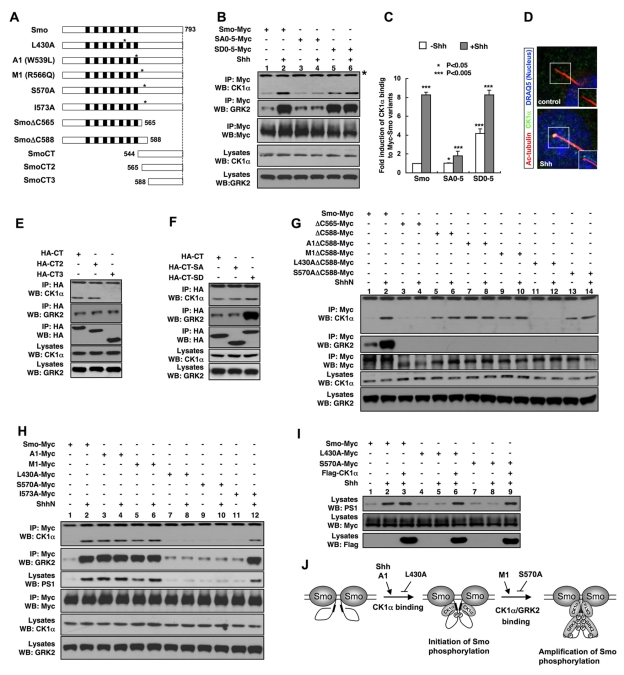
Shh promotes CK1α/GRK2 binding to Smo. (A) Schematic drawings of full-length and truncated Smo with point mutations indicated by the asterisks. Black boxes denote the transmembrane domains. L430A is located in the third intracellular loop; A1 in the seventh transmembrane domain; M1, S570A, and I573A are located in the membrane proximal region of the Smo C-tail. (B–C, E–H) Coimmunoprecipitation assays to determine the interaction between CK1α/GRK2 with different forms of Smo. NIH 3T3 cells were transfected with the indicated Myc-tagged or HA-tagged Smo constructs, followed by immunoprecipitation and western blot analysis with indicated antibodies. Cell lysates were also directly immunoblotted by the indicated antibodies. Histogram in (C) shows the quantification of (B) with CK1α binding normalized by the Smo input and compared with lane 1. *p<0.05, **p<0.01, ***p<0.005. Western blots were quantified using the ImageJ software followed by Prism analysis, *n* = 3. Quantifications of (G) and (H) are shown in Figure S5. (D) NIH 3T3 cells untreated (control) or treated with Shh-conditioned medium were immunostained to show the expression of acetylated tubulin (red), CK1α (green), and DRAQ5 (blue). Images in the insets are shifted overlays of the selected fields. (I) NIH 3T3 cells were transfected with the indicated Myc-tagged Smo constructs either alone or together with a Flag-tagged CK1α construct and treated with or without Shh-conditioned medium, followed by immunoprecipitation and western blot analysis with the indicated antibodies. Cell lysates were also directly immunoblotted by the indicated antibodies. (J) A model for how Smo phosphorylation is regulated by Shh. See text for details.

Finally, we investigated how Shh induces Smo phosphorylation by testing the possibility that Shh promotes the accessibility of Smo to its kinases. By immunoprecipitation assay, we found that Shh markedly increased the association between Smo-Myc and endogenous CK1α and GRK2 in NIH3T3 cells (Figure 7B, lanes 1–2; Figure 7C). In addition, Shh induced accumulation of CK1α in primary cilia (Figure 7D). The binding of CK1α/GRK2 to Smo-Myc is specific because we did not detect association between Smo-Myc and endogenous CK1ε or GRK5 under the same condition (unpublished data).

To further explore the interactions between Smo and CK1α/GRK2 and their regulation, we generated several N- or C-terminally truncated forms of Smo (Figure 7A). As shown in Figure 7E, CK1α and GRK2 coimmunoprecipitated with HA-tagged Smo C-tail from aa 544 to aa 793 (SmoCT). Deletion of aa 544–565 from the Smo C-tail (SmoCT2) did not affect CK1α/GRK2 binding; however, further deletion of aa 565–588 (SmoCT3) abolished CK1α binding but did not affect GRK2 binding, suggesting that the membrane proximal region of Smo C-tail between aa 565 and 588 mediates CK1α binding, whereas the distal region between aa 588 and 793 binds GRK2. Consistent with this, we found that SmoΔC588 but not SmoΔC565 pulled down CK1α and neither SmoΔC565 nor SmoΔC588 pulled down GRK2 (Figure 7G, lanes 3–6). Thus, the CK1α binding pocket is located N-terminal to the phosphorylation sites.

Interestingly, SmoΔC588 exhibited increased basal binding to CK1α (Figure 7G, compare lanes 1 and 5), suggesting that the distal region of Smo C-tail inhibits CK1α binding in the absence of Shh. We hypothesized that unphosphorylated Smo C-tail adopts a closed conformation that could mask the membrane proximal CK1α binding domain (Figure 7J). Indeed, the SA0–5 mutation, which locked Smo C-tail in its closed conformation, diminished Shh-stimulated CK1α binding, whereas the SD0–5 mutation, which locked the Smo C-tail in its open conformation, increased the basal CK1α binding (Figure 7B, lanes 3–6; Figure 7C). However, the SA0–5 and SD0–5 mutations in the context of Smo C-tail (SmoCT-SA and SmoCT-SD) did not significantly alter CK1α binding (Figure 7F), unlike their effect in the context of full-length Smo. Thus, instead of directly altering the CK1α binding site, phosphorylation may regulate CK1α binding by influencing the conformation of Smo C-tail and thus controlling the accessibility of the CK1α binding pocket. In contrast, the SD0–5 mutation dramatically increased GRK2 binding in the context of both SmoCT and full-length (Figure 7B and 7F), suggesting that phosphorylation may increase the affinity of a GRK2 binding site(s) in the Smo C-tail.

Although kinase binding to Smo is influenced by phosphorylation, we found that Shh still enhanced the binding of CK1α to SmoSA0–5 and SmoSD0–5 (Figure 7B, lanes 3–6; Figure 7C). Furthermore, CK1α binding to SmoΔC588, which lacks all the CK1/GRK phosphorylate sites, was also upregulated by Shh (Figure 7G, compare lanes 5 and 6; Figure S5). These results demonstrate that Shh can stimulate CK1α binding through a phosphorylation-independent mechanism. In contrast, GRK2 binding to SmoSD0–5 or SmoSA0–5 was no longer regulated by Shh (Figure 7B, lanes 3–6), suggesting that Shh promotes GRK2 binding mainly through the phosphorylation-dependent mechanism. Taken together, these data suggest that Shh may regulate CK1α/GRK2 binding in two steps: 1) Shh stimulates CK1α binding to Smo prior to its phosphorylation, which may provide a mechanism to initiate Smo phosphorylation, and 2) phosphorylation of Smo C-tail releases its inhibition on CK1α binding and at the same time increases its binding affinity for GRK2, leading to amplification of Smo phosphorylation (Figure 7J).

### Regulation of CK1α Binding and Smo Phosphorylation by Gain- or Loss-of-Function Smo Mutations

To establish the relationship between kinase association and Smo phosphorylation, we examined how gain- or loss-of-function Smo mutations affect CK1α binding, including two oncogenic mutations (A1 and M1) and three loss-of-function mutations in or near the CK1α binding pocket identified by previous studies (Figure 7A) [28],[39]. We found that both A1 and M1 resulted in a constitutive CK1α/GRK2 binding and Smo phosphorylation with A1 being more potent than M1 (Figure 7H, lanes 3 and 5; Figure S5). In addition, Shh further increased the binding of CK1α to and phosphorylation of SmoM1 but not SmoA1 (Figure 7H, lanes 4 and 6; Figure S5). In contrast, the loss-of-function mutations L430A and S570A blocked Shh-induced CK1α/GRK2 binding and Smo phosphorylation (Figure 7H, lanes 7–10; Figure S5). Another loss-of-function mutation, I573A, which mainly affected Smo stability [28], slightly reduced Shh-stimulated CK1α binding and Smo phosphorylation (Figure 7H, lanes 11–12; Figure S5).

If L430A and S570A affect Smo phosphorylation because they interfere with the accessibility of Smo to its kinases, one would expect that increasing the levels of Smo kinases might rescue the phosphorylation defect. Indeed, cotransfection of CK1α with SmoL430A or SmoS570A resulted in their efficient phosphorylation (Figure 7I).

Finally, we found that A1 mimicked Shh stimulation to enhanced CK1α binding to SmoΔ588 (Figure 7G, compare lanes 7–8 with 5–6; Figure S5), whereas L430A blocked both the basal and Shh-stimulated binding of CK1α to SmoΔ588 (Figure 7G, lanes 11–12, Figure S5), suggesting that these mutations affect the phosphorylation-independent mechanism that regulates CK1α binding (Figure 7J). It is possible that the third intracellular loop may also contribute to CK1α binding and this is disrupted by L430A.

In contrast, the M1 and S570 mutations did not affect either the basal or Shh-stimulated binding of CK1α to SmoΔ588 (Figure 7G, lanes 9–10 and 13–14; Figure S5). Thus, M1 and S570 affect CK1α binding only in the context of full-length Smo and may act mainly by regulating the release of C-tail inhibition (Figure 7J).

## Discussion

Smo is a central component of the Hh signal transduction cascade and an important cancer drug target, but the molecular mechanism by which Smo is activated has remained poorly understood. In this study, we demonstrate that Smo is activated by multi-site phosphorylation mediated by CK1α and GRK2, and phosphorylation promotes both ciliary localization and active conformation of Smo. We provide evidence that graded Shh signals induce increasing levels of Smo phosphorylation that fine-tune Smo activity. In addition, we demonstrate that oncogenic mutations and small molecule Hh pathway modulators including SAG, oxysterols, and cyclopamine regulate Smo through CK1α/GRK2-mediated phosphorylation. We provide evidence that Shh promotes Smo phosphorylation by regulating its accessibility to CK1α/GRK2 and effective Smo phosphorylation depends on the primary cilium. The CK1α/GRK2 sites we identified are conserved among vertebrate Smo proteins; thus, the mechanism we uncover here is likely to be conserved in other vertebrate species.

### CK1α and GRK2 Regulate Smo Through Multi-Site Phosphorylation

It has been well established that *Drosophila* Smo is hyperphosphorylated by multiple kinases in response to Hh stimulation [22],[23],[25]–[27]; however, sequence divergence between *Drosophila* and vertebrate Smo proteins makes it unclear whether vertebrate Smo proteins are similarly phosphorylated in response to Hh. Using the phospho-tag gel and a phospho-specific antibody, we provide the first evidence that Shh induces hyperphosphorylation of Smo, which is mediated by CK1α and GRK2. Several lines of evidence suggest that CK1α and GRK2 are bona fide Smo kinases. First, our in vitro kinase assay with purified Smo fragments and recombinant kinases demonstrated that both CK1 and GRK phosphorylate multiple sites in Smo C-tail. Second, mutating the CK1/GRK sites in the Smo C-tail abolished Shh-stimulated Smo phosphorylation in vivo. Third, using a phospho-specific antibody that recognized an overlapping CK1/GRK site (S1), we demonstrated that Shh induced phosphorylation at this site through CK1α and GRK2.

We identified a total of six CK1α/GRK2 phosphorylation regions, which we named S0 to S5. S0 and S1 contain multiple phospho-acceptor Ser/Thr residues. Our functional study suggests that S0 and S1 play a major role while other sites play a fine-tuning role in Smo regulation. The employment of multi-site phosphorylation may allow graded Hh morphogens to induce different levels of Smo activity through differential phosphorylation. Indeed, we found that increasing levels of Shh induced a progressive increase in the level of Smo phosphorylation. Furthermore, increasing the number of SA mutations gradually decreased the level of Shh-induced Smo activity, whereas increasing the number of phospho-mimetic mutations progressively increased the level of basal Smo activity.

Although phospho-mimetic mutations increase the basal activity of Smo both in vitro and in vivo, they do not confer full activation of Smo, which is in contrast to the A1/M2 oncogenic mutation. One possibility is that the SD mutations may not fully mimic phosphorylation and may even lock Smo in a less optimal conformation for activation. However, we think this is unlikely because the SmoSD variants can be further stimulated by Shh to reach their full activity. In addition, phospho-mimetic mutations did not affect SmoA1 activity (unpublished data). These observations suggest that Shh and A1/M2 may stimulate an additional mechanism(s) that acts in conjunction with CK1α/GRK2-mediated phosphorylation to fully activate Smo. The proposed paralleled mechanisms could be phosphorylation-independent and/or could involve additional kinase(s). Furthermore, although our in vitro and in vivo assays suggest that phosphorylation at S0–S5 is mediated by CK1α/GRK2, we cannot rule out the possibility that some of these sites might also be phosphorylated by other kinases.

### Smo Phosphorylation, Conformational Switch, and Ciliary Localization

A prevalent view regarding Smo activation is that Hh activates Smo by inducing its ciliary localization [16],[17]. However, this view has been challenged by more recent studies showing that the Smo inhibitor cyclopamine promotes instead of blocks ciliary localization of Smo [18],[19],[45], suggesting that ciliary localization of Smo is insufficient for its activation. Our previous and current studies demonstrate that Shh induces a conformational switch in Smo that is also induced by the A1 mutation and SAG but is blocked by Smo inhibitors including cyclopamine [22],[46],[47], suggesting that Hh-induced Smo conformational switch may represent an additional step for Smo activation. How Smo conformational switch and ciliary localization are regulated remained unknown. Here we demonstrate that both events are governed by CK1α/GRK2-mediated phosphorylation of Smo C-tail. CK1α/GRK2 phosphorylation-deficient forms of Smo are locked in a closed conformation and fail to accumulate in primary cilia in response to Shh stimulation, whereas phospho-mimetic forms adopt an open conformation and accumulate in the primary cilia independent of Shh.

In the absence of Hh, Smo may move in and out of the primary cilium with the exit rate far exceeding the entry rate, resulting in a low steady state level of Smo in the primary cilium. However, Hh-induced Smo phosphorylation and conformational change could tilt the balance by increasing the entry rate and/or decreasing the exit rate. In support of this model, we found that Hh-induced phosphorylation promoted the binding of β-arr2 to Smo. A recent study demonstrated that β-arrestins mediate the interaction between Smo and the anterior-grade trafficking motor kinesin-II [43]. Thus, Hh-induced phosphorylation may promote Smo ciliary accumulation by facilitating its anterior grade trafficking through recruiting β-arr2. It is also possible that phosphorylation may impede the retrograde trafficking of Smo or may stabilize Smo protein in the primary cilium.

### Regulation of Smo Phosphorylation

Our data suggested that Shh stimulates Smo phosphorylation, at least in part by regulating the accessibility of Smo to its kinases. Our deletion analyses revealed that CK1α and GRK2 bind Smo through the membrane proximal and distal regions of Smo C-tail, respectively. We provided evidence that Smo C-tail in its closed conformation inhibits CK1α binding likely by masking the membrane proximal CK1α binding pocket through steric hindrance, and this inhibition is released by phosphorylation that promotes the open conformation of Smo C-tail. Furthermore, we demonstrate that Shh stimulates the binding of CK1α to the membrane proximal region of Smo C-tail through a mechanism that parallels with the phosphorylation-dependent mechanism. We propose a two-step mechanism for Shh-regulated kinase association and Smo phosphorylation (Figure 7J). In the first step (referred to as the initiation step), Shh stimulates CK1α binding to Smo prior to its phosphorylation, likely by inducing a local conformational change near the membrane proximal region that either optimizes the CK1α binding pocket or makes it more accessible to CK1α. This step may contribute to the initiation of Smo phosphorylation and is promoted by the A1 mutation but is blocked by the L430A mutation. In the second step (referred to as the amplification step), CK1α-initiated phosphorylation further increases CK1α binding by promoting the open conformation of Smo C-tail. Furthermore, phosphorylation of Smo C-tail increases its binding affinity for GKR2. Increased binding of CK1α/GRK2 forms a feedback loop to further increase the level of Smo phosphorylation.

There could be a basal association of CK1α/GRK2 with Smo in quiescent cells, and Shh could induce a change in Smo that makes the CK1/GRK sites more accessible to the bound kinases, which may also contribute to the initiation of Smo phosphorylation. Finally, Smo phosphorylation is likely to be counteracted by a phosphatase(s), which could be essential for keeping basal Smo phosphorylation low. Therefore, Shh could regulate the activity or accessibility of a Smo phosphatase(s) in addition to regulating the Smo kinases.

Our time course study revealed that phosphorylation of Smo occurred more rapidly in the primary cilia compared with the whole cell (Figure 6). In addition, expression of a dominant negative form of Kif3b, which blocks ciliogenesis, attenuated Shh- or A1-induced Smo phosphorylation. These observations suggest that Smo phosphorylation occurs more efficiently in the primary cilia. Interestingly, we found that CK1α is accumulated in primary cilia in response to Shh stimulation (Figure 7D). The increase in the local concentration of CK1α may explain, at least in part, why phosphorylation of Smo is more effective in the primary cilium. It is also possible that Shh-mediated inhibition of Ptc is more effective in the primary cilium.

### Parallels Between Mammalian and *Drosophila* Smo Activation

**Figure 8:**
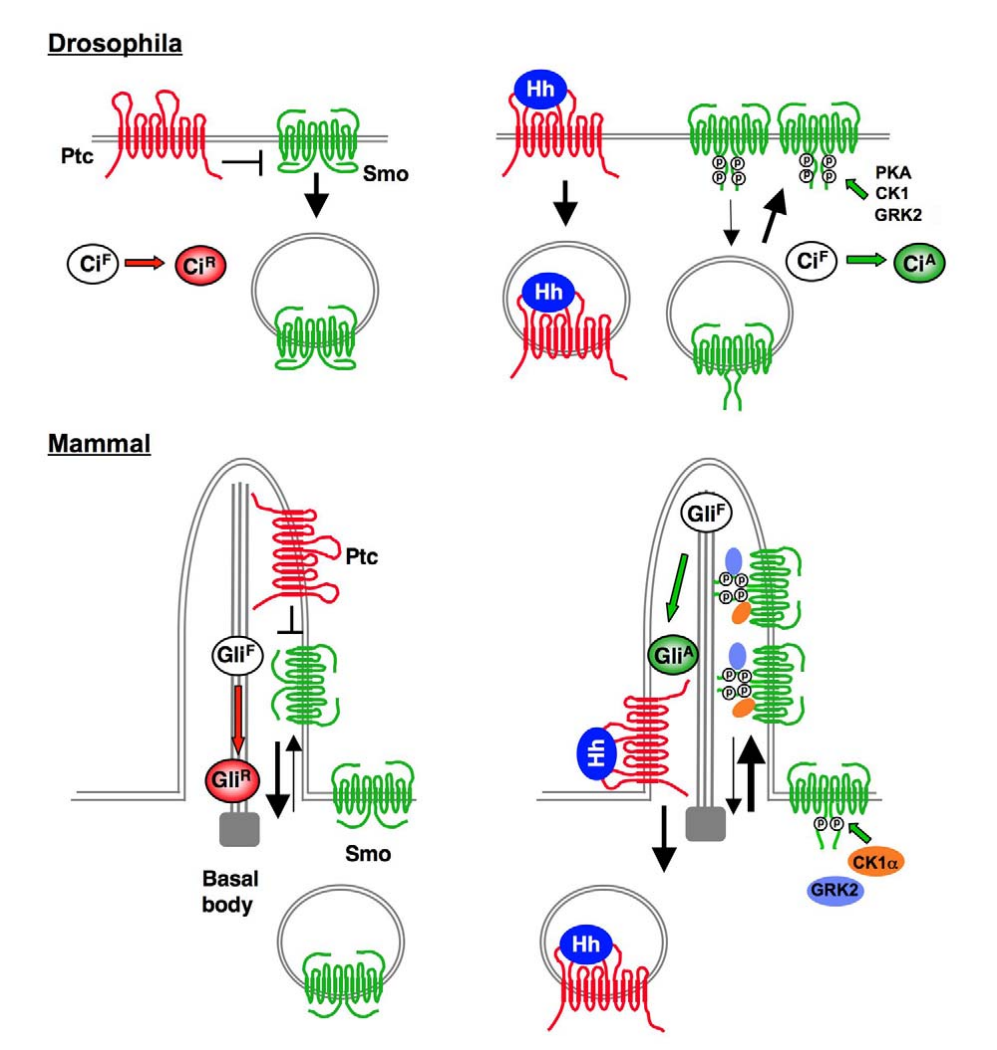
A unified mechanism for Smo activation in different species. Multi-site phosphorylation by distinct but overlapping sets of kinases activates mSmo and dSmo by regulating their subcellular localization and conformation. See text for details.

Despite the profound difference in the primary sequence between *Drosophila* and vertebrate Smo, our study suggests that their activation mechanisms are remarkably similar (Figure 8). In both cases, Hh induces Smo phosphorylation at multiple sites (although by distinct sets of kinases) that fine-tune Smo activity, and phosphorylation activates Smo by inducing its active conformation and regulating its subcellular localization (cell surface accumulation for dSmo and ciliary accumulation for mSmo). Hh-stimulated phosphorylation induces dSmo conformation change by antagonizing multiple Arg clusters in its C-tail [22]. As the inactive conformation of mSmo is also maintained by a long stretch of basic cluster in its C-tail [22], multisite phosphorylation may promote mSmo conformational change through a similar mechanism. A recent study has demonstrated that GRK2 regulates dSmo by both kinase-dependent and kinase-independent mechanisms [48]. The observation that Shh induces mSmo/GRK2 complex formation raises an interesting possibility that GRK2 may also function as a molecular scaffold to promote mSmo activation.

## Materials and Methods

### Constructs

pGE-Smo-CFP^C^, pGE-Smo-YFP^C^, and pGE-Smo-CFP^L2^YFP^C^ have been described previously [22]. SmoSA and SmoSD substitutions were generated by site-directed PCR mutagenesis. To generate GST-Smo fusion constructs, DNA fragments encoding Smo C-terminal regions with wild type or mutated phosphorylation sites were amplified by PCR and inserted between *SalI* and *NotI* sites of the *pGEX-4T-3* vector. To construct XZ201-Smo-CFP retrovirus, Smo-CFP variants were PCR out and subcloned between *HpaI* and *SalI* sites in the MSCV retroviral vector (XZ201, gift from Dr. Alec Zhang's lab in UTSW). The bovine source of kinase-expressing constructs used in the shRNA rescue experiments were generated by PCR amplification and cloned into pCDNA3.1(+) vector, the dominant negative form of bovine GRK2 (bGRK2-K220R) was generated by site-directed PCR mutagenesis strategy, and the pCS2(+)-CK1α and pCS2(+)-DN-CK1α are gifts from John Graff's Lab [49]. LMP/shRNA against kinase: CK1α, GRK2, or GRK5 were constructed by inserting indicated shRNA fragments into LMP vector (Open Biosystems) containing a PGK-puromycin resistance-IRES-GFP cassette. To generate HA-tagged wild type, SA0–5 or SD0–5 versions of Smo C-tail, wild type, or mutant DNA fragments were amplified by PCR and inserted between *NotI* and *XbaI* sites in the *HA-pUAST* vectors [50], and the HA-tagged constructs were subcloned into *pCDNA3.1(+)* vector with *EcoRI* and *XbaI* sites. All the constructs were sequence verified. DN-Kif3b constructs were kindly provided by Dr. Pao-Tien Chuang [44].

### In Vitro Kinase Assay

CK1/GRK in vitro kinase assay was performed according to the manufacturer's instruction (Upstate Biotechnologies, 14-714). Briefly, GST-fusion proteins, 0.1 mM ATP containing 10 mCi of γ-^32^p-ATP and kinases: CK1δ (New England Biolabs), GRK5 (Upstate Biotechnologies, 14-714), were mixed well and incubated at 30°C for 1.5 h in reaction buffer (20 mM Tris-HCl, pH 8.0, 2 mM EDTA, 10 mM MgCl2, 1 mM DTT); the reactions were stopped by adding 4× SDS loading buffer and boiled at 100°C for 5 min; and the phosphorylation of GST-fusion proteins were analyzed by autoradiography after SDS-PAGE.

### Cell Cultures

Unless otherwise noted, all the mammalian cell lines were cultured in DMEM, supplemented with 10% fetal bovine serum (FBS), L-glutamine, 1 mM sodium pyruvate, and penicillin. NIH 3T3 cells were obtained from ATCC. *smo* ^−*/*−^ and *Kif3a* ^−*/*−^ mouse embryonic fibroblasts were kindly provided by Dr. Pao-Tien Chuang [44]. Wild type MEFs were derived from wild type mice embryos at 9.5 dpc, embryos were dissected to pieces and transferred to 10 cm dishes for adherence, regular DMEM medium were slowly added, fibroblasts cells that migrated from the embryos were collected by trypsinization after 3∼5 d, and expanded wild-type MEFs were aliquot and frozen for further use. Reagents were used in the following concentrations unless otherwise noted: Recombinant Mouse Sonic Hedgehog N-terminus (ShhNp, R&D systems, Cat #464-SH), 293-Shh-conditioned medium (1∶6 v/v; [40]), SAG (200 nM), cyclopamine (1 µM), CKI-7 (10 µM; Sigma), and Heparin (1 µM; Sigma). SAG and cyclopamine are gifts from Dr. James Chen at Stanford University. The kinase inhibitors were added into the medium the night before collecting the samples, and for heparin treatment, 5 µg/ml Lipofectin (Invitrogen) were mixed together with the medium to facilitate their entry into the cells.

### Transfection, Immunoprecipitation, Western Blot, Immunochemistry, and FRET

For protein expression, cells were transfected with FuGENE 6 transfection reagent (Roche) according to the manufacturer's instructions, harvested and lysed in RIPA buffer (50 mM Tris-Cl at pH 7.9, 150 mM NaCl, 5 mM EDTA), 1% NP-40 supplemented with protease inhibitors (Roche), and lysates were frozen and thaw 2∼3 times. Immunoprecipitation experiments were performed as previously described [51]. The Phos tag-conjugated SDS-PAGE analysis was performed according to the standard protocols [34]. Phos tag-conjugated acrylamide was purchased from the NARD Institute in Japan. First and secondary antibodies used in this study: mouse anti-Myc (1∶5,000; Sigma), rabbit anti-Myc (A-14; Santa Cruz Biotechnologies), mouse anti-HA (1∶10,000; Santa Cruz Biotechnologies), mouse anti-Flag (1∶10,000; Santa Cruz Biotechnologies), rabbit anti-CK1α (Santa Cruz Biotechnologies), rabbit anti-GRK2 (Santa Cruz Biotechnologies), rabbit anti-GRK5 (Santa Cruz Biotechnologies), rabbit phospho-specific antibodies against S1 (PS1, 1∶50), monoclonal anti-Acetylated tubulin (1∶1,000; Sigam#T7451), Goat anti-mouse IgG HRP (1∶10,000), and Goat anti-rabbit IgG HRP (1∶10,000). PS1 antibody was generated by Genemed Synthesis Inc., phosphorylated peptide EP(pS)ADV(pSpS)AWAQHVTC was injected into rabbit, the serum was affinity-purified by antigen, and the flow-through from the affinity-purification was also kept as control antibody S1 against non-phosphorylated peptide. For immunofluorescence, cells were seeded on ploy-D Lysine coated LAB-TEK chamber slides and were transfected with indicated constructs, followed by treating with indicated reagents for indicated time. Cells were washed 2 times with 1XPBS and fixed with 4% PFA, permeabilized, stained, and mounted for observation with Zeiss LSM510 confocal microscope. FRET assays were performed essentially as previously described [22]. Briefly, CFP was exited at 458 nM wavelength and YFP at 514 nM wavelength. CFP signals were collected once before photobleaching (BP) and once after photobleaching (AP) of YFP. YFP was photobleached with full power of the 514 nM laser line for 1∼2 min at the top half of the cells, leaving the bottom half as an internal control. The CFP signals from the bleached half (both membrane and cytoplasmic signals) were used for FRET calculating, and the efficiency of FRET was calculated with the formula: FRET%  =  [(CFP_AP_ − CFP_BP_)/CFP_AP_] ×100.

### *8XGliBS-Luciferase* Assay

The day before transfection, different cell lines were seeded at a density of 1∼2×10^5^ cells/ml in 24-well plates, and cells were transfected with *8XGliBS-luciferase* and *pRL-TK* at 4∶1 ratio, and 5% w/w of pGE-Smo constructs with Fugene 6 (Roche) according to the manufacturer's instructions. After 2 d of transfection, cells were changed to low serum medium (DMEM supplemented with 0.5% calf serum) with or without Shh-conditioned medium combined with additional treatments as indicated, and cells were harvested and luciferase activities were determined using the Dual Luciferase Reporter Assay System (Promega) and FLUOstar OPTIMA (BMGLABTCH). Each sample was performed in triplicate and the assays were repeated for at least 3 times.

### Chick In Ovo Electroporation

All constructs were electroporated into the neural tube of HH st11–12 chick embryos [52]. Embryos were harvested 48 h after electroporation, fixed, and processed for immunohistochemistry as previously described [53]. The following antibodies were used: mouse Pax7, Nkx6.1, Nkx2.2 (from DSHB), rabbit Olig2 (Chemicon), rabbit Islet1/2 (a gift from Dr. T. Jessell), and GFP (Biogenesis). Anterior thoracic levels were analyzed in all cases.

### Retroviral Infection and shRNA

Stable NIH 3T3/shRNA cell lines against kinase CK1α, GRK2, or GRK5 were generated by retroviral infection and selected with 3 µg/ml of puromycin.

HEK 293T cells were transfected with XZ201 retrovirus vectors encoding variant Smo cDNAs and pCL-Eco packaging vector, and supernatants were collected 72 h post-transfection, filtered through a 0.45 µM syringe filter, and added to 50∼70% confluent wild type MEFs with 8 µg/ml polybrene (Sigma) overnight.

## Supporting Information

Figure S1 CK1α and GRK2 regulate Smo phosphorylation and Shh signaling activity. (A) Diagrams showing the sequences of the corresponding shRNAs targeting CK1α, GRK2, or GRK5. (B) Knockdown efficiency by the indicated shRNAs. (Top) Cell extracts were prepared from NIH3T3 cells with integrated LMP control vector (CT) or vectors expressing shRNA against different regions of CK1α, GRK2, or GRK5 and immunoblotted with CK1α, GRK2, GRK5, and GAPDH antibodies. Representative western blots were repeated 3 to 5 times. The intensity of each band was analyzed using the ImageJ software. The numbers indicated percentage of knockdown. (Bottom) Knockdown efficiency of individual stable NIH 3T3/shRNA lines measured by real-time PCR. (C–E) Stable NIH 3T3/shRNA lines were transfected with Smo and WT or dominant-negative (DN) bovine CK1α (bCK1α) or GRK2 (bGRK2) together with the *8XGliBS-luc* reporter and control *pRL-TK* construct, and treated with or without Shh-conditioned medium, followed by dual Luciferase assay. (F) Cell extracts from stable NIH 3T3/shRNA cell lines or control NIH 3T3 cells transfected with Smo-Myc and treated with or without Shh-conditioned medium were separated on Phos tag-conjugated SDS-PAGE gel and probed with Myc antibody. (G) *Gli-luciferase* assay in NIH 3T3 cells in response to Shh stimulation or kinase overexpression. (H) *Gli-luciferase* assay in control or CK1α/GRK2 shRNA expressing NIH 3T3 cells treated with or without Shh-conditioned medium.
(TIF)

Figure S2 CK1 and GRK phosphorylate multiple sites in Smo. (A–C) CK1 and GRK phosphorylate individual serine in the S1 site. (A) A schematic drawing full-length Smo with the sequences for S1, S2, and S3 indicated underneath. Amino acid substitutions for individual constructs are indicated. (B–C) In vitro kinase assay using recombinant CK1δ (B) or GRK5 (C) and purified GST-Smo608–670 fusion proteins with wild type (WT) sequence or indicated substitutions. (D–E) CK1/GRK sites in Smo C-tail mediate Smo activation by Shh, CKIα, GRK2, and GRK5. (D) *Gli-luc* assay in NIH 3T3 cells transfected with Smo or SmoSA0–5 with or without the indicated kinase expressing constructs and treated with or without Shh-conditioned medium. (E) FRET analysis in NIH 3T3 cells transfected with Smo-CFP^C^/YFP^C^ or SmoSA0–5-CFP^C^/YFP^C^ with or without the indicated kinase expressing constructs and treated with or without Shh-conditioned medium (mean ± s.d., *n*≥10). (F) Evaluation of the specificity of the PS1 antibody. (Top) A schematic drawing of full-length Smo with the antigen peptide sequence for generating the PS1 antibody indicated. (Bottom) Western blot analysis using the PS1 antibody or antibodies against the non-phosphorylated peptide (S1). The PS1 antibody recognized GST-Smo608–670 but not GST-Smo608–670SA phosphorylated by GRK or CK1. In addition, the PS1 antibody did not recognize the unphosphorylated GST-Smo608–670. Equal amounts of GST fusion proteins were loaded as indicated by western blot with the S1 antibody. (G) Knockdown of CK1α or GRK2 affected Shh-induced Smo phosphorylation. Cell extracts from indicated stable NIH 3T3/shRNA lines transfected with Smo-Myc and treated with or without Shh-conditioned medium were separated on SDS-PAGE gel and immunoblotted with PS1 and Myc antibodies. Knockdown of CK1α or GRK2 but not GRK5 reduced Shh-induced Smo phosphorylation at S1 site. (H–I) The effect of mutating CK1/GRK sites on A1-induced Smo phosphorylation and Smo activity in response to Shh and small molecules. (H) Cell extracts from NIH 3T3 cells transfected with Smo-Myc, SmoA1-Myc, or SmoA1SA1–5-Myc and treated with or without Shh-conditioned medium were separated on Phos tag-conjugated SDS-PAGE gel and immunoblotted with a Myc antibody. Mutating multiple CK1/GRK sites abolished Shh- or A1-induced mobility shift of the Myc-tagged Smo. (I) *Gli-luc* assay in NIH 3T3 cells transfected with Smo, SmoSA0–5, or SmoSD0–5 and treated with or without the indicated reagents. The activity of SmoSA0–5 was no longer induced by Shh or SAG, whereas SmoSD0–5 exhibited elevated basal activity and was more resistant to cyclopamine (CYC) inhibition.
(TIF)

Figure S3 Mutating CK1/GRK sites affect Smo activity in chick neural tube. (A) Activity of Smo SD variants in chick neural tube. SmoWT, SmoSD1, SmoSD12, SmoSD123, SmoSD1–5, or SmoSD0–5 were transfected by in ovo electroporation into the thoracic region of HH st11–12 chick neural tube and the expression patterns of the indicated markers analyzed 48 h later. In embryos transfected with SmoSD123, SmoSD1–5, or SmoSD0–5, the expression of Pax7 was repressed and expression of Isl1, Olig2, and Nkx2.2 expanded dorsally (arrows). By contrast, the expression patterns of the neural tube markers in SmoSD1 or SmoSD12 electroporated embryos were similar to those in embryos transfected with SmoWT. (B) Mutating S1 affects SmoA1 activity in chick neural tube. SmoA1 or SmoA1 with different combination of SA mutations (A1SA1, A1SA12, A1SA13, A1SA23, and A1SA123) were transfected by in ovo electroporation into the thoracic region of the neural tube of HH st11–12 chick embryos and the expression patterns of the indicated markers analyzed 48 h later. SmoA1 exhibited constitutive signaling activity, resulting in the dorsal expansion of ventral markers, including Islet1, Nkx6.1, Olig2, and Nkx2.2 and the repression of Pax7 (Brackets). Mutating S1 alone (A1SA1) or in combination with other sites (A1SA12, A1SA13, or A1SA123) markedly reduced the signaling activity of SmoA1 and these constructs only induced mild ectopic expression of ventral markers (arrows). By contrast, mutating S2 and S3 (A1SA23) did not significantly affect SmoA1 activity.
(TIF)

Figure S4 Primary cilium and Smo phosphorylation. (A) Quantification of Smo-CFP or PS1 positive cilia in NIH 3T3^Smo-CFP^ treated with different reagents. NIH 3T3^Smo-CFP^ cells were either untreated or treated with Shh-conditioned medium (Shh), SAG (200 nM), 20-OHC (10 µM), CYC (10 µM), or a combination of Shh and CYC (10 µM), SAG (200 nM) and CYC (10 µM), or 20-OHC (10 µM) and CYC (10 µM). The histogram indicates the percentage of Smo-CFP or PS1 positive cilia. Over 100 ciliated cells were counted for each time point (*n* = 3). (B) FRET analysis in wild type or *Kif3a*−*/*− MEFs transfected with Smo-CFP^C^/YFP^C^, SmoA1-CFP^C^/YFP^C^, or SmoSD0–5-CFP^C^/YFP^C^ and treated with or without Shh-conditioned medium (mean ± s.d., *n*≥10). (C) *Gli-luc* assay in *Kif3a*−*/*− MEFs transfected with the indicated constructs and treated with or without Shh-conditioned medium.
(TIF)

Figure S5 Quantification of CK1α binding to different forms of Smo. Histograms for the western blot analyses shown in Figure 7G and Figure 7H. The pull-downed CK1α signal intensity in each lane was normalized by the pull-downed Smo signal intensity and compared with lane 1. *p<0.05, **p<0.01, ***p<0.005. The signal intensity for each band was quantified by ImageJ software followed by Prism analysis, *n* = 3.
(TIF)

## Abbreviations

20-OHC: 20α-hydroxycholesterol
CYC: cyclopamine
dSmo: *Drosophila* Smoothened
FRET: fluorescence resonance energy transfer
GFP: green fluorescent protein
GPCR: G protein coupled receptor
GRK: G protein coupled receptor kinase
mSmo: mammalian Smoothened
PAGE: polyacrylamide gel electrophoresis
PBS: phosphate buffed saline
PKA: protein kinase A
PP: λ phosphatase
SDS: sodium dodecyl sulfate
Shh: Sonic hedgehog
