# Supplementary figures and images for: Sonic Hedgehog Dependent Phosphorylation by CK1α and GRK2 Is Required for Ciliary Accumulation and Activation of Smoothened
Source: PLoS Biol. 2011 Jun 14;9(6):e1001083. doi: 10.1371/journal.pbio.1001083 (PMC3114773; doi:10.1371/journal.pbio.1001083)

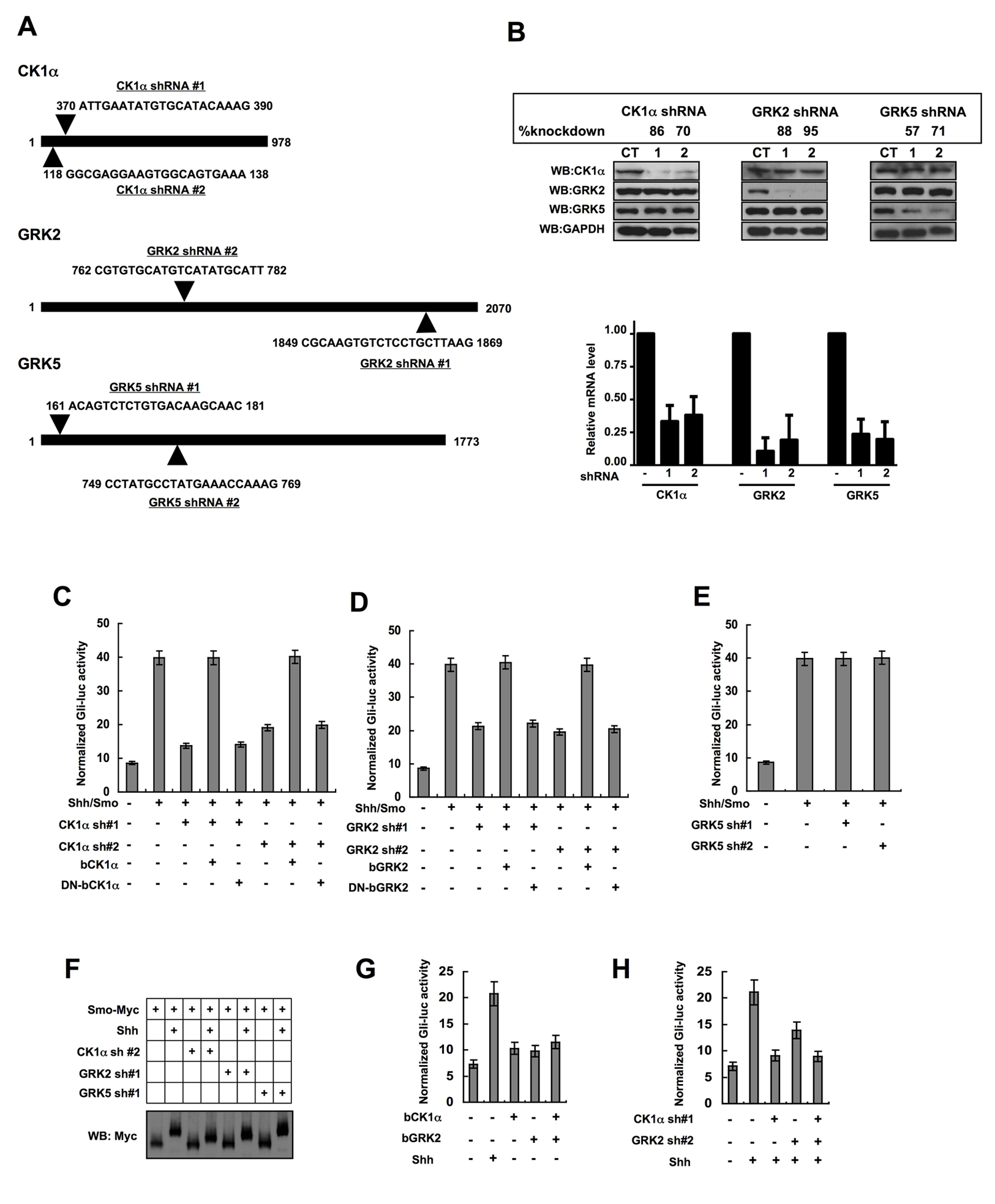

Supplement: Figure S1 — CK1α and GRK2 regulate Smo phosphorylation and Shh signaling activity. (A) Diagrams showing the sequences of the corresponding shRNAs targeting CK1α, GRK2, or GRK5. (B) Knockdown efficiency by the indicated shRNAs. (Top) Cell extracts were prepared from NIH3T3 cells with integrated LMP control vector (CT) or vectors expressing shRNA against different regions of CK1α, GRK2, or GRK5 and immunoblotted with CK1α, GRK2, GRK5, and GAPDH antibodies. Representative western blots were repeated 3 to 5 times. The intensity of each band was analyzed using the ImageJ software. The numbers indicated percentage of knockdown. (Bottom) Knockdown efficiency of individual stable NIH 3T3/shRNA lines measured by real-time PCR. (C–E) Stable NIH 3T3/shRNA lines were transfected with Smo and WT or dominant-negative (DN) bovine CK1α (bCK1α) or GRK2 (bGRK2) together with the 8XGliBS-luc reporter and control pRL-TK construct, and treated with or without Shh-conditioned medium, followed by dual Luciferase assay. (F) Cell extracts from stable NIH 3T3/shRNA cell lines or control NIH 3T3 cells transfected with Smo-Myc and treated with or without Shh-conditioned medium were separated on Phos tag-conjugated SDS-PAGE gel and probed with Myc antibody. (G) Gli-luciferase assay in NIH 3T3 cells in response to Shh stimulation or kinase overexpression. (H) Gli-luciferase assay in control or CK1α/GRK2 shRNA expressing NIH 3T3 cells treated with or without Shh-conditioned medium. (TIF) [file pbio.1001083.s001.tif]

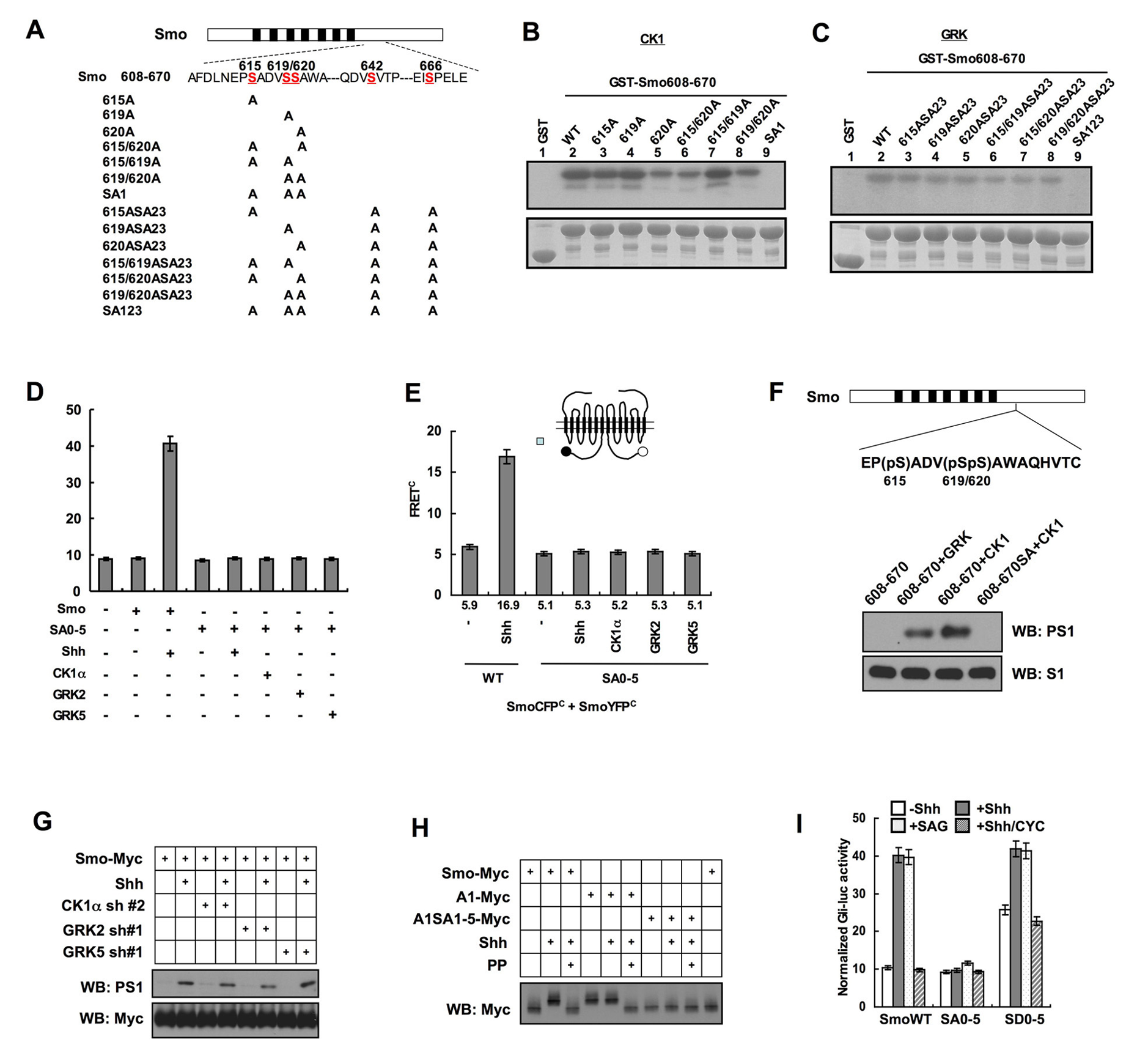

Supplement: Figure S2 — CK1 and GRK phosphorylate multiple sites in Smo. (A–C) CK1 and GRK phosphorylate individual serine in the S1 site. (A) A schematic drawing full-length Smo with the sequences for S1, S2, and S3 indicated underneath. Amino acid substitutions for individual constructs are indicated. (B–C) In vitro kinase assay using recombinant CK1δ (B) or GRK5 (C) and purified GST-Smo608–670 fusion proteins with wild type (WT) sequence or indicated substitutions. (D–E) CK1/GRK sites in Smo C-tail mediate Smo activation by Shh, CKIα, GRK2, and GRK5. (D) Gli-luc assay in NIH 3T3 cells transfected with Smo or SmoSA0–5 with or without the indicated kinase expressing constructs and treated with or without Shh-conditioned medium. (E) FRET analysis in NIH 3T3 cells transfected with Smo-CFPC/YFPC or SmoSA0–5-CFPC/YFPC with or without the indicated kinase expressing constructs and treated with or without Shh-conditioned medium (mean ± s.d., n≥10). (F) Evaluation of the specificity of the PS1 antibody. (Top) A schematic drawing of full-length Smo with the antigen peptide sequence for generating the PS1 antibody indicated. (Bottom) Western blot analysis using the PS1 antibody or antibodies against the non-phosphorylated peptide (S1). The PS1 antibody recognized GST-Smo608–670 but not GST-Smo608–670SA phosphorylated by GRK or CK1. In addition, the PS1 antibody did not recognize the unphosphorylated GST-Smo608–670. Equal amounts of GST fusion proteins were loaded as indicated by western blot with the S1 antibody. (G) Knockdown of CK1α or GRK2 affected Shh-induced Smo phosphorylation. Cell extracts from indicated stable NIH 3T3/shRNA lines transfected with Smo-Myc and treated with or without Shh-conditioned medium were separated on SDS-PAGE gel and immunoblotted with PS1 and Myc antibodies. Knockdown of CK1α or GRK2 but not GRK5 reduced Shh-induced Smo phosphorylation at S1 site. (H–I) The effect of mutating CK1/GRK sites on A1-induced Smo phosphorylation and Smo activity in response to Shh and sma [file pbio.1001083.s002.tif]

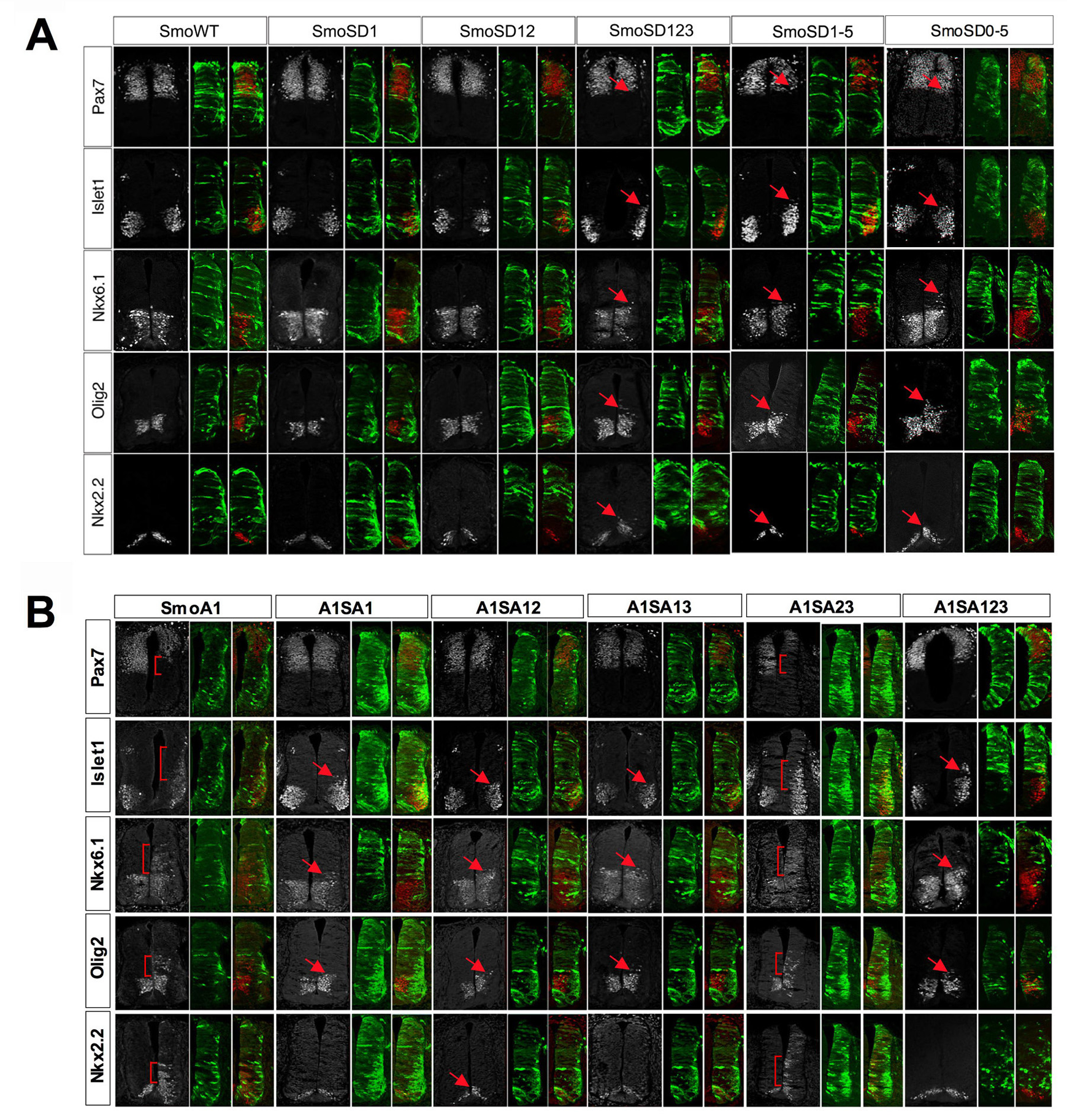

Supplement: Figure S3 — Mutating CK1/GRK sites affect Smo activity in chick neural tube. (A) Activity of Smo SD variants in chick neural tube. SmoWT, SmoSD1, SmoSD12, SmoSD123, SmoSD1–5, or SmoSD0–5 were transfected by in ovo electroporation into the thoracic region of HH st11–12 chick neural tube and the expression patterns of the indicated markers analyzed 48 h later. In embryos transfected with SmoSD123, SmoSD1–5, or SmoSD0–5, the expression of Pax7 was repressed and expression of Isl1, Olig2, and Nkx2.2 expanded dorsally (arrows). By contrast, the expression patterns of the neural tube markers in SmoSD1 or SmoSD12 electroporated embryos were similar to those in embryos transfected with SmoWT. (B) Mutating S1 affects SmoA1 activity in chick neural tube. SmoA1 or SmoA1 with different combination of SA mutations (A1SA1, A1SA12, A1SA13, A1SA23, and A1SA123) were transfected by in ovo electroporation into the thoracic region of the neural tube of HH st11–12 chick embryos and the expression patterns of the indicated markers analyzed 48 h later. SmoA1 exhibited constitutive signaling activity, resulting in the dorsal expansion of ventral markers, including Islet1, Nkx6.1, Olig2, and Nkx2.2 and the repression of Pax7 (Brackets). Mutating S1 alone (A1SA1) or in combination with other sites (A1SA12, A1SA13, or A1SA123) markedly reduced the signaling activity of SmoA1 and these constructs only induced mild ectopic expression of ventral markers (arrows). By contrast, mutating S2 and S3 (A1SA23) did not significantly affect SmoA1 activity. (TIF) [file pbio.1001083.s003.tif]

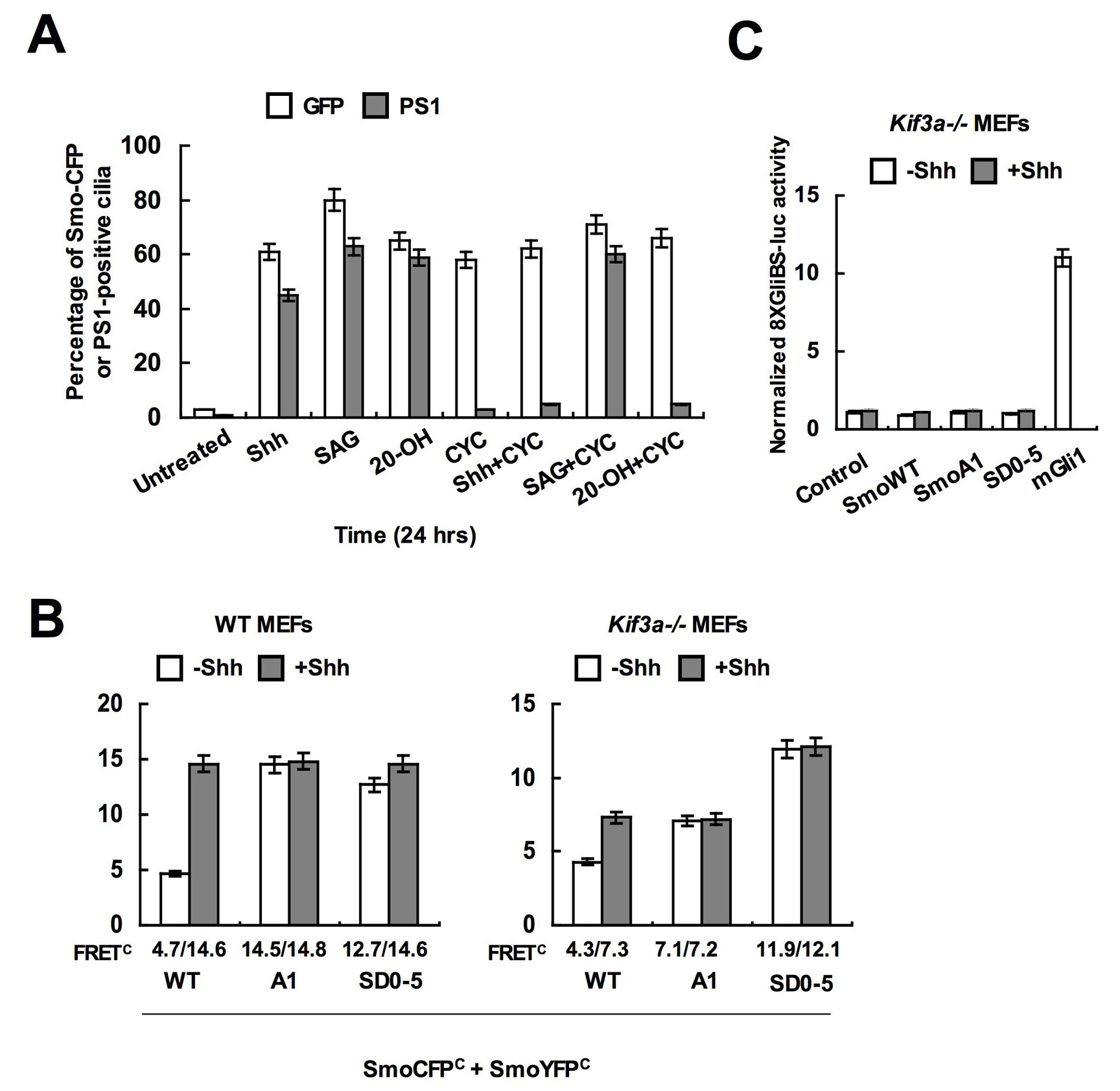

Supplement: Figure S4 — Primary cilium and Smo phosphorylation. (A) Quantification of Smo-CFP or PS1 positive cilia in NIH 3T3Smo-CFP treated with different reagents. NIH 3T3Smo-CFP cells were either untreated or treated with Shh-conditioned medium (Shh), SAG (200 nM), 20-OHC (10 µM), CYC (10 µM), or a combination of Shh and CYC (10 µM), SAG (200 nM) and CYC (10 µM), or 20-OHC (10 µM) and CYC (10 µM). The histogram indicates the percentage of Smo-CFP or PS1 positive cilia. Over 100 ciliated cells were counted for each time point (n = 3). (B) FRET analysis in wild type or Kif3a−/− MEFs transfected with Smo-CFPC/YFPC, SmoA1-CFPC/YFPC, or SmoSD0–5-CFPC/YFPC and treated with or without Shh-conditioned medium (mean ± s.d., n≥10). (C) Gli-luc assay in Kif3a−/− MEFs transfected with the indicated constructs and treated with or without Shh-conditioned medium. (TIF) [file pbio.1001083.s004.tif]

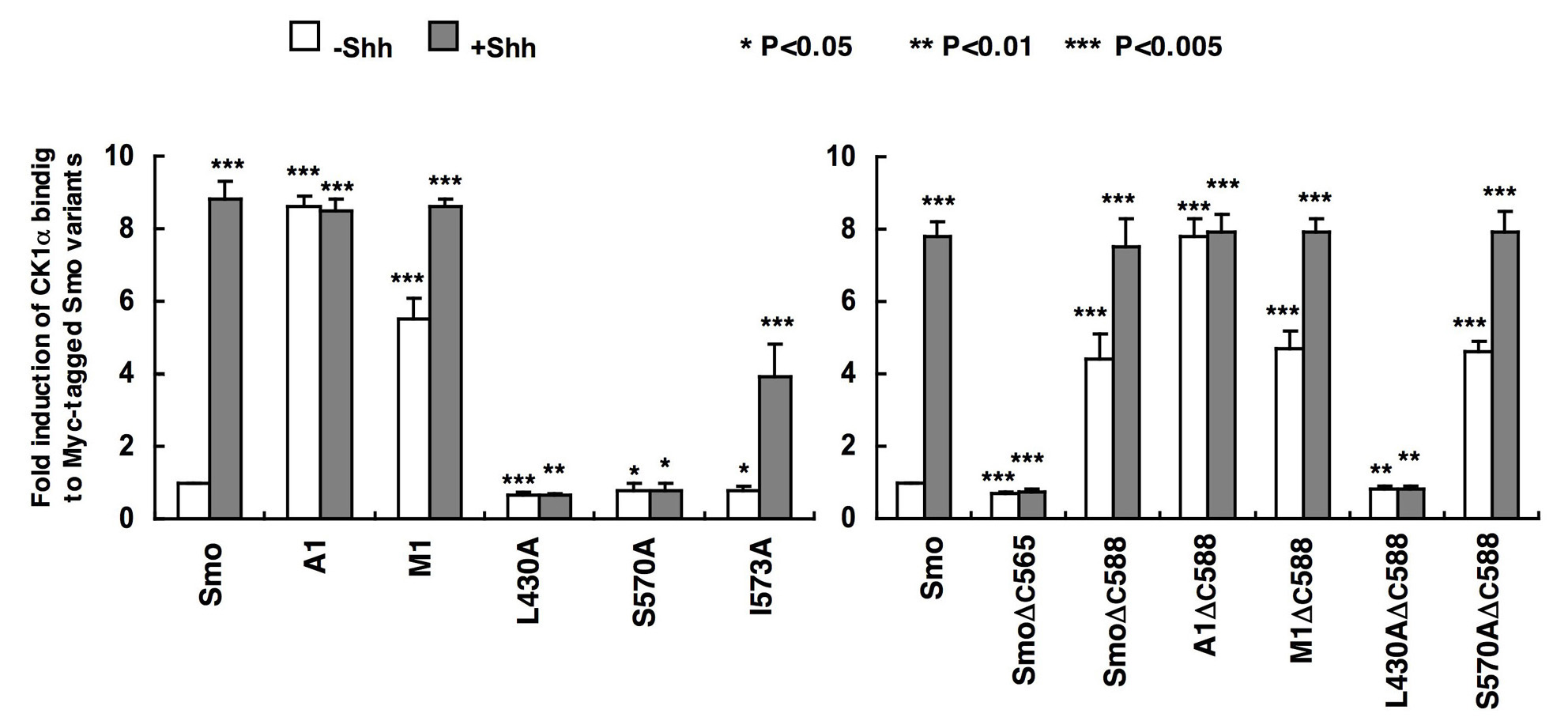

Supplement: Figure S5 — Quantification of CK1α binding to different forms of Smo. Histograms for the western blot analyses shown in Figure 7G and Figure 7H. The pull-downed CK1α signal intensity in each lane was normalized by the pull-downed Smo signal intensity and compared with lane 1. *p<0.05, **p<0.01, ***p<0.005. The signal intensity for each band was quantified by ImageJ software followed by Prism analysis, n = 3. (TIF) [file pbio.1001083.s005.tif]
